# The role of polyproline motifs in the histidine kinase EnvZ

**DOI:** 10.1371/journal.pone.0199782

**Published:** 2018-06-28

**Authors:** Magdalena Motz, Kirsten Jung

**Affiliations:** Center for Integrated Protein Science Munich at the Department of Biology I, Microbiology, Ludwig-Maximilians-Universität München, Martinsried, Germany; Centre National de la Recherche Scientifique, Aix-Marseille Université, FRANCE

## Abstract

Although distinct amino acid motifs containing consecutive prolines (polyP) cause ribosome stalling, which necessitates recruitment of the translation elongation factor P (EF-P), they occur strikingly often in bacterial proteomes. For example, polyP motifs are found in more than half of all histidine kinases in *Escherichia coli* K-12, which raises the question of their role(s) in receptor function. Here we have investigated the roles of two polyP motifs in the osmosensor and histidine kinase EnvZ. We show that the IPPPL motif in the HAMP domain is required for dimerization of EnvZ. Moreover, replacement of the prolines in this motif by alanines disables the receptor’s sensor function. The second motif, VVPPA, which is located in the periplasmic domain, was found to be required for interaction with the modulator protein MzrA. Our study also reveals that polyP-dependent stalling has little effect on EnvZ levels. Hence, both polyP motifs in EnvZ are primarily involved in protein-protein interaction. Furthermore, while the first motif occurs in almost all EnvZ homologues, the second motif is only found in species that have MzrA, indicating co-evolution of the two proteins.

## Introduction

Proline differs from all other natural amino acids in possessing a pyrrolidine ring, a five-membered ring that includes the amino group. This chemical structure fixes the torsional angle ɸ of the N-C_α_ bond and restricts conformational flexibility [1]. Due to its exceptional rigidity, proline is not only a poor substrate for the ribosomal peptidyl transferase reaction, but induces kinks and acts as an α-helix breaker in proteins [2, 3]. Proline is the sole amino acid that can adopt *cis* and *trans* conformations [4]. Thus, a sequence of consecutive prolines results in the formation of either the right-handed poly (*cis*-) proline helix I (PPI) or the left-handed poly (*trans*-) proline helix II (PPII). PPII is accepted to be the third major secondary structure element in folded proteins and is often involved in protein- and nucleic-acid-binding sites [5–7].

Translation of two or more consecutive prolines causes ribosomes to stall until translation elongation factor P (EF-P) binds to the ribosome and alleviates the arrest [3, 8–11]. Bacteria have developed various unique post-translational modification systems for EF-P that are required for its function at stalling sites, which underlines the importance of this elongation factor [12–14]. Similarly, the eukaryotic eIF5A and archaeal aIF5A, which are orthologous to EF-P, have an essential function in these organisms [11, 15–18]. Although virtually all di-proline-containing motifs cause translational stalling, the duration of stalling is modulated by amino acids located upstream and downstream of the arrest motif [8, 19, 20]. For example, amino acids like Cys or Thr preceding a three-proline motif attenuate the arrest, whereas Arg and His promote it [20]. Therefore, polyP motifs are defined as a consecutive stretch of prolines with flanking residues: X_(-2)_X_(-1)_-nP-X_(+1)_, n≥2; where X_(-2)_, X_(-1)_ and X_(+1)_ can be any other amino acid. We recently classified these motifs according to their stalling efficiency into strong, medium and weak motifs [21]. Although EF-P alleviates stalling, formation of the Pro-Pro bond is markedly slower [22, 23]. Therefore, polyP motifs are disfavored during evolution [21]. Nevertheless, about 10% of all proteins in the *E*. *coli* K-12 proteome include polyP motifs implying that their benefits must outweigh their maintenance. Among these proteins, 18 of the 30 histidine kinases (HKs) in *E*. *coli* K-12 carry at least one polyP motif, and 8 of those (BaeS, CreC, CpxA, EnvZ, EvgS, QseC, PhoR and RcsD) have a strong stalling motif.

**Fig 1:**
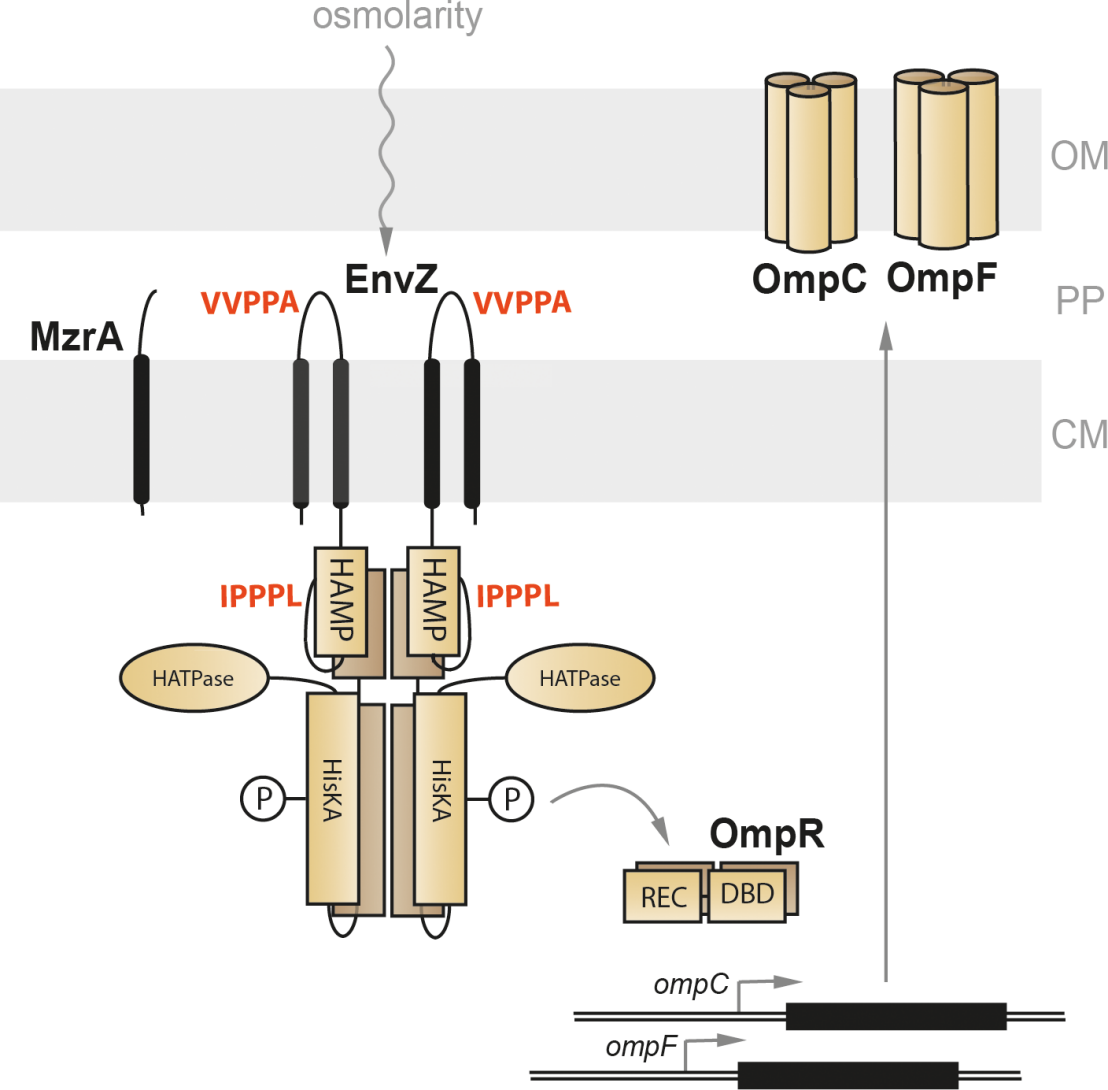
Schematic overview of the EnvZ/OmpR signaling cascade. EnvZ is located in the cytoplasmic membrane and senses alterations in osmolarity. It transduces the signal via phosphorylation to its cognate response regulator OmpR, which in turn reciprocally adjusts the expression of the target genes *ompC* and *ompF*, which code for outer membrane porins with different pore diameters. MzrA also resides in the cytoplasmic membrane and modulates the activity of EnvZ. PolyP motifs and their localization are shown in red. CM–cytoplasmic membrane; PP–periplasm; OM–outer membrane.

This raises the question of the functional role of polyP motifs in these sensors. Three consecutive prolines form part of the active center in the universally conserved Val-tRNA synthetase ValS and are essential for efficient charging of the tRNA with valine and prevention of mischarging with threonine [24]. The membrane-integrated pH sensor and transcriptional activator CadC contains two polyP motifs. As a consequence, the copy number of CadC is extremely low, and this feature was found to be crucial for stringent control of expression of its target genes [9]. A recent systemic analysis of the distribution and localization of polyP motifs in proteins proposes that they might be important for co-translational folding and/or membrane insertion [21]. Here we focus on the role of polyP motifs in EnvZ, a representative of the family of sensor histidine kinases. The dimeric histidine kinase EnvZ in *E*. *coli*, together with OmpR, responds to osmotic, but also to acid stress [25–35]. EnvZ is anchored in the cytoplasmic membrane by two transmembrane helices, which flank a periplasmic domain (Fig 1). The C-terminal cytoplasmic part of EnvZ comprises three distinct domains, HAMP, HisKA and HATPase [29, 36]. Although the periplasmic domain is assumed to be involved in stimulus perception, recent studies show that the cytoplasmic portion can sense intracellular alterations resulting from extracellular osmotic changes [31–33, 37]. EnvZ is bifunctional, acting as a kinase under conditions of high external osmolarity, and as a phosphatase at low osmotic pressures [38]. Phosphorylated OmpR reciprocally regulates transcription of *ompC* and *ompF*, which code for two outer membrane porins with different pore sizes (reviewed in [39]) (Fig 1).

The signaling activity of EnvZ is modulated by MzrA, an integral cytoplasmic membrane protein, which interacts with EnvZ via its periplasmic domain [40] (Fig 1). In doing so, MzrA functionally connects the EnvZ/OmpR system with the stress-response systems CpxA/CpxR and σ^E^ [40, 41].

EnvZ has two polyP motifs: one strong stalling motif (IPPPL) in the cytoplasmic HAMP domain (amino acids 201–205; designated here as polyP_c_), and one medium-to-weak stalling motif (VVPPA) in the periplasmic domain (amino acids 71–75; polyP_p_) (Fig 1). Our data indicate that both motifs are required for protein-protein interactions, promoting either the homodimerization of EnvZ or its association with MzrA. In addition, we present evidence indicating that the polyP_p_ motif in EnvZ has co-evolved with MzrA. Hence, we conclude that both polyP motifs in EnvZ have essentially structural rather than strictly regulatory functions.

## Material and methods

### Media and growth conditions

*E*. *coli* strains were cultivated in LB [1% (w/v) tryptone, 0.5% (w/v) yeast extract, 171 mM NaCl] or M9 medium (45 mM Na_2_HPO_4_·2H_2_O, 22 mM KH_2_PO_4_, 8.5 mM NaCl, 18.7 mM NH_4_Cl, 2 mM MgSO_4_, 22 mM glucose, 0.1 mM CaCl_2_) at 37°C under aerobic growth conditions if not stated otherwise.

To test the effects of osmotic stress, M9 medium was supplemented with NaCl (0.2 or 0.4 M) or sucrose (0.4 or 0.8 M). The osmolality of these media was measured with an osmometer (Gonotec, Osmomat 030). For selection purposes, antibiotics were added at concentrations of 50 μg/ml (kanamycin) and 100 μg/ml (ampicillin).

### Site-directed mutagenesis of *envZ*

Proline-to-alanine replacements were introduced into the chromosomally encoded *envZ* of the *E*. *coli* strains MG1655 *rpsL150* [42] and EPB273a ([27] kindly provided by Mark Goulian, University of Pennsylvania) in two steps. (i) Marker-less deletion of the native *envZ* locus was achieved by using the pRED/ET system and a removable kanamycin cassette flanked by FRT sites with 50-bp homology arms (Quick & Easy *E*. *coli* Gene Deletion by Red^®^/ET^®^ Recombination kit from Gene Bridges) [43]. (ii) *envZ* fragments with codon substitutions leading to the replacement of consecutive prolines by alanines were generated by overlap PCR and cloned into the vector pNPTS138-R6KT [44]. The resulting plasmid was then transferred into the *envZ* deletion mutants by conjugation with the donor *E*. *coli* strain WM3064 (kindly provided by William Metcalf, University of Illinois). Homology arms (500 bp long) flanking the plasmid-encoded *envZ* gene mediated its chromosomal integration by homologous recombination at the native locus. The plasmid backbone was removed via counter-selection as previously described [44]. The resulting mutations were verified by PCR and sequencing. Primer sequences used for plasmid and strain construction will be provided on request.

### Preparation of outer membrane proteins

*E*. *coli* cells were grown to mid-exponential phase in 200 ml of M9 medium supplemented with NaCl or sucrose depending on the experiment, harvested by centrifugation at 4°C and 4500 *x g* for 30 min and resuspended in Tris/HCl buffer [20 mM Tris/HCl, pH 7.5, 150 mM NaCl, 10% (v/v) glycerol, 1 mM DTT, 0.5 mM PMSF]. All subsequent preparation steps were performed at 4°C. After high-pressure cell disruption, cell debris was removed by centrifugation as described above. Membrane vesicles were then prepared by ultracentrifugation of the supernatant at 250,000 *x g* for 45 min. The proteins of the cytoplasmic membrane were solubilized by resuspension of the pellet in 2 ml of Na-phosphate buffer (10 mM, pH 7.2) containing 2% (w/v) Triton X-100 and incubation at 37°C for 1 h. The suspension was then centrifuged at 390,000 *x g* for 30 min. The supernatant was discarded, and the pellet was washed in Na-phosphate buffer without Triton and centrifuged again at 390,000 *x g* for 30 min. The final pellet was resuspended in 100 μl PBS (8.1 mM Na_2_HPO_4_, 1.47 mM KH_2_PO_4_, 137 mM NaCl, 2.68 mM KCl) and SDS sample buffer was added [final concentration: 50 mM Tris/HCl pH 6.8, 2% (w/v) SDS, 0.1% (w/v) bromophenol blue, 10% (v/v) glycerol, 100 mM DTT]. Samples were then supplemented with 25 mg urea and heated at 100°C for 5 min before gel electrophoresis (5 μl per lane).

### SDS-PAGE

Proteins were fractionated on a 12.5% (w/v) SDS polyacrylamide gel (acrylamide:bis-acrylamide 37.5:1). Concentrated outer membrane proteins were fractionated on a 10% SDS polyacrylamide gel (acrylamide:bisacrylamide 44:1) supplemented with 4 M urea [45]. Gels were stained with Coomassie Blue.

### Quantitative Western blots

Proteins were transferred onto PVDF membranes by wet blotting. EnvZ was detected with specific anti-EnvZ antibodies (kindly provided by Linda Kenney, National University of Singapore), or anti-FLAG antibodies (abcam) diluted 1:5,000 in TBS-T buffer [10 mM Tris/HCl pH 7.5, 150 mM NaCl, 0.05% (v/v) Tween 20] supplemented with 0.75% (w/v) skim-milk powder. Bound antibodies were detected with alkaline-phosphatase-conjugated anti-rabbit or anti-mouse antibodies (Rockland), diluted to 1:4,000 in TBS-T buffer. After developing the blot with NBT/BCIP (0.175 mg/ml BCIP, 0.225 mg/ml NBT, 50 mM Na_2_CO_3_/NaHCO_3_ pH 9.5), the membrane was scanned and the bands quantitatively analyzed using the software ImageJ.

### Porin translation assay

This assay is based on the previously published method [46]. Reporter strains *E*. *coli* EPB273a EnvZ_WT_ and EPB273a EnvZ_P/A(c)_ were grown overnight in M9 medium. This culture was then used to inoculate fresh M9 medium, and cells were grown to mid-exponential phase. Cultures were diluted 1:500 in fresh M9 medium supplemented with NaCl or sucrose depending on the experiment. Aliquots (200 μl) of each culture were transferred to Greiner 96-well plates and incubated under constant agitation at 37°C to an OD_600_ of about 0.2. After a 15-min incubation on ice, fluorescence was measured with a TECAN Reader (infinite 200Pro, program: Tecan i-control).

### Bacterial two-hybrid assay

The *mzrA* or *envZ* coding sequence was fused to the 5’ end of pKT25 or pT18 (Euromedex) encoding the corresponding, complementing adenylate cyclase fragments [47, 48]. We used four different variants of *envZ*: the wild-type gene, and *envZ* with codon substitutions that converted the VVPPL motif into VVAAL, or IPPPL into IAAAL, or both replacements together.

For the analysis of protein-protein interactions, we used the *E*. *coli* reporter strain BTH101 [49], which was transformed with combinations of pT18-EnvZ plus pT25-EnvZ or pT18-EnvZ plus pT25-MzrA variants. LB overnight cultures were diluted 1:500 in 2-ml aliquots of fresh LB medium and incubated at 37°C for 2 h, followed by induction with 0.5 mM IPTG. Cells were incubated for an additional 12 h and harvested. β-Galactosidase activity was determined as described before [9]. In parallel, 5-μl aliquots of cells grown in LB medium to an OD_600_ of 0.2 were plated on LB agar supplemented with 1 mM IPTG and 40 μg/ml 5-bromo-4-chloro-3-indolyl-β-D-galactopyranoside (X-Gal). After incubation at 30°C for 24 h, galactosidase activity was quantified based on the intensity of the blue color of the colonies.

### Modelling of the three-dimensional structure of the EnvZ HAMP domain

The full-length EnvZ sequence from *E*. *coli* K-12 was downloaded from the UniProt database (UniProt Entry: P0AEJ4) and used as the template for modelling of its 3-D structure with Phyre 2.0 [50]. For the purposes of illustration, the HAMP domain was visualized separately with the software Chimera [51].

### Alignment and construction of phylogenetic trees

To identify non-redundant EnvZ orthologues, we carried out a BLAST search of the UniProt Microbial Proteomes database using the full-length EnvZ from *E*. *coli* K-12 as the query sequence (expect value:1, auto matrix, allowed gaps). Sequences shorter than 90% of the *E*. *coli* K-12 EnvZ sequence were excluded. A pairwise alignment of 793 sequences was done with a progressive algorithm from the software CLC Workbench 7.6 (CLC Bio Qiagen, Hilden, Germany), using the following parameters: gap open cost 10, gap extension cost 1, high accuracy. The results served as the basis for construction of a phylogenetic tree by the software’s high-accuracy, distance-based neighbor-joining algorithm (100 bootstrap replicates and the Jukes-Cantor distance correction as default parameters). We screened these organisms for MzrA by searching for orthologues of *E*. *coli* K-12 MzrA with NCBI Protein BLAST (blastp algorithm; expect threshold 10; matrix BLOSUM62).

In addition, we selected both *E*. *coli* K-12 EnvZ and MzrA orthologues (also identified by NCBI protein BLAST) from the Gammaproteobacteria included in the tree of life recently published by Hug *et al*. [52]. The tree was visualized with iTOL [53, 54]. To analyze amino acid conservation, we aligned all 63 EnvZ sequences using the CLC Workbench, as described above.

## Results

### EnvZ harbors a polyP motif (IPPPL) in its HAMP domain

**Fig 2:**
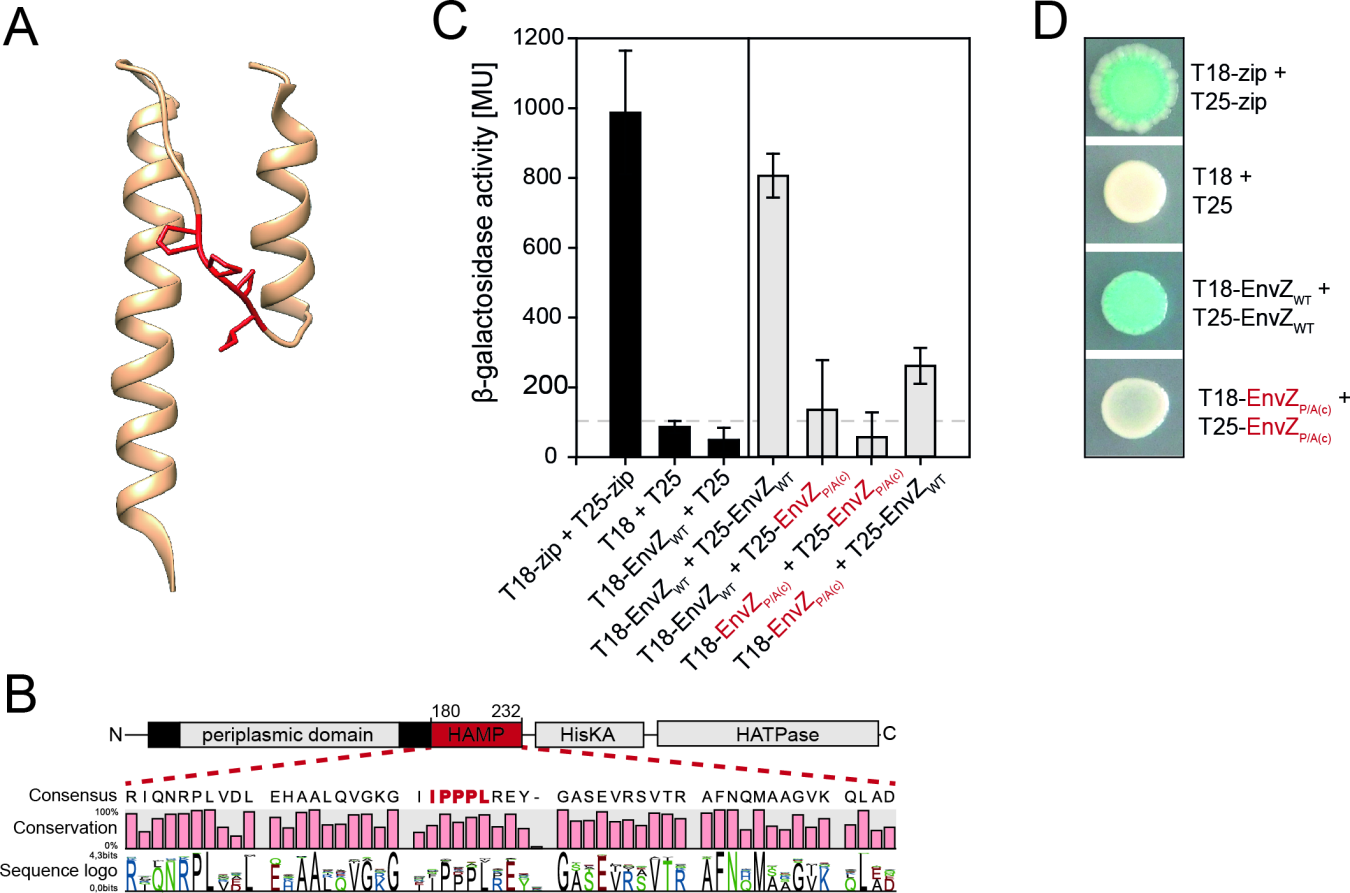
The role of polyP_c_ in EnvZ dimerization. (A) The 3D structure of the HAMP domain of EnvZ in *E*. *coli* K-12, modelled with Phyre. Proline residues of the polyP_c_ motif are marked in red. (B) Sequence conservation of the EnvZ HAMP domain based on the alignment of 63 EnvZ homologues (exhibiting >44% sequence identity to *E*. *coli* K-12 EnvZ) from a phylogenetic tree of representative Gammaproteobacteria [52]. (C) Two-hybrid analysis (BACTH assay) of the significance of EnvZ polyP_c_ for EnvZ dimerization, based on the complementation of T25 and T18 adenylate cyclase fragments fused N-terminally to EnvZ. The histograms depict β-galactosidase activities after transformation of the reporter strain BTH101 with plasmids encoding the indicated hybrids. Cells were grown in LB medium and harvested 12 h after induction with IPTG. The data is based on biological triplicates, and error bars indicate standard deviations of the mean. (D) Determination of β-galactosidase activity of BTH101 cells, transformed with plasmid encoded T18/T25-EnvZ variants, as revealed by blue staining of colonies grown on X-Gal/IPTG agar plates for 24 h. The experiment was repeated three times and a representative plate for each interaction condition is shown.

*E*. *coli* EnvZ contains an IPPPL motif in the HAMP domain (polyP_c_), which is thought to cause strong translational arrest [21]. Using the structural prediction software tool Phyre 2 [50], we localized the IPPPL motif within the unstructured connector region between the two α-helices characteristic of the HAMP domain (Fig 2A). The degree of conservation of protein motifs indicates their relative importance for protein functionality. Therefore, we analyzed the external node organisms of a recently published phylogenetic species tree [52] for orthologues of the *E*. *coli* K-12 EnvZ. Among the Gammaproteobacteria, we found 63 species that have EnvZ (proteins that show 44–100% sequence identity to the EnvZ from *E*. *coli* K-12). Sequence alignment revealed strong conservation specifically of the prolines in the IPPPL motif: Ile_201_ 60%, Pro_202_ 89%, Pro_203_ 68%, Pro_204_ 81%, Leu_205_ 87% (Fig 2B and S1 Fig). Taking into account the fact that polyP motifs are under selection pressure, the degree of conservation of these amino acids suggests that they have an important function [21]. Thus the polyP motifs themselves, or the translation pause they induce, might play a role in domain folding or membrane insertion [21]. However, the IPPPL motif in EnvZ neither separates two domains nor is it located at an appropriate distance from a transmembrane helix. In addition, the amino acid upstream of the three prolines, which primarily determines the strength of the motif [20], is only 60% conserved. Isoleucine is often replaced by phenylalanine, which generally weakens the stalling strength of the motif [20]. *A priori*, this suggests that the prolines themselves may be of greater consequence than the duration of the ribosomal stalling they may cause.

### PolyP_c_ is required for EnvZ dimerization

HAMP domains form a homodimeric, four-helical, parallel coiled-coil structure and are crucial for signal transduction of receptor proteins [55–58], but not all include a polyP motif [59]. To test whether the polyP_c_ motif is important for dimerization of EnvZ, we used a bacterial two-hybrid (BACTH) assay, which is based on the split adenylate cyclase (CyaA) from *Bordetella pertussis* [47, 60]. We compared the dimerization capacity of wild-type EnvZ with that of a variant in which the prolines of the polyP_c_ motif were replaced by alanines. The assay is based on the (dimerization-dependent) complementation of the adenylate cyclase fragments (T25 and T18), which are translationally fused to the cytoplasmic N-terminal ends of EnvZ. Functional reassembly of the adenylate cyclase induces a cAMP signalling cascade, which activates transcription of the *lac* operon in the *E*. *coli* reporter strain BTH101 [49]. As a positive control, we showed dimerization of the transcription factor GCN4 (Zip), and as negative control, we confirmed that the T18 and T25 fragments alone do not interact (Fig 2C). As expected, combination of the two wild-type EnvZ fusions resulted in high β-galactosidase activities, confirming dimerization of the protein. Replacement of the IPPPL motif resulted in an EnvZ variant that was unable to dimerize (Fig 2C). A major loss in dimerization was already seen when the motif was replaced in either the T18 or T25 monomer. These results were confirmed by comparing the blue color of colonies of the corresponding strains cultivated on IPTG/X-Gal LB-agar plates (Fig 2D).

### Effect of the polyP_c_ motif on the response of EnvZ to osmotic stress

To further investigate the importance of the IPPPL motif for the signaling activity of EnvZ, we replaced it with IAAAL in the chromosomally encoded EnvZ and characterized the mutant’s response to osmotic stress by measuring in two distinct ways the ability of EnvZ to regulate the expression of *ompC* and *ompF* via OmpR.

**Fig 3:**
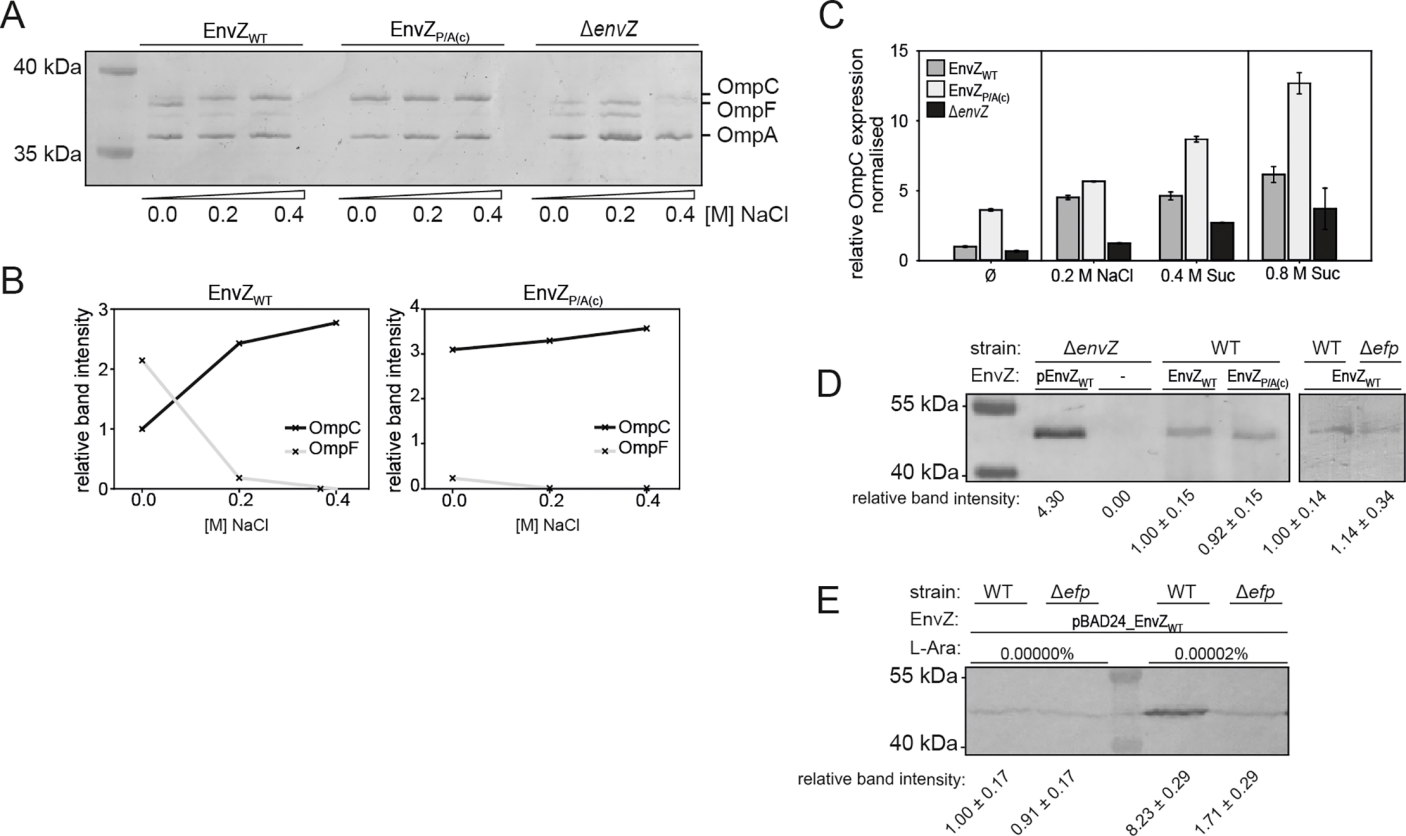
The role of polyP_c_ in EnvZ function. An *E*. *coli* wild-type control (EnvZ_WT_), the *envZ* deletion strain (Δ*envZ*) and the mutant in which the IPPPL motif was replaced by IAAAL (EnvZ_P/A(c)_) were characterized. (A) Analysis of EnvZ/OmpR target gene expression in response to osmotic stress caused by the addition of 0.2 or 0.4 M NaCl to the growth medium (M9 medium). Cells were grown to the mid-exponential growth phase. Outer membrane proteins were isolated from *E*. *coli* K-12 EnvZ_WT_, and the EnvZ_P/A(c)_ and Δ*envZ* mutants, fractionated on an SDS-urea gel and stained with Coomassie Blue. The experiment was repeated three times and a representative gel is shown. (B) Quantification of OmpF and OmpC band intensities of the gel is shown in (A). (C) Levels of OmpC-CFP fluorescence were measured in *E*. *coli* EPB273a [27] reporter strains deleted for *envZ* (*ΔenvZ*) or expressing wild-type EnvZ or the EnvZ_P/A(c)_ variant, following exposure to osmotic stress imposed by added NaCl or sucrose (Suc) for approximately 3 hours (OD_600_ = 0.2). The addition of 0.2 M NaCl and 0.4 M sucrose, respectively, corresponded to an increase in the medium osmolality from 0.2 to 0.460 Osmol/kg. In the presence of 0.8 M sucrose the medium osmolarity was determined with 1.080 Osmol/kg. The results are based on the analysis of biological triplicates and values were normalized to the fluorescence level of wild-type EnvZ grown in M9 medium (value 1.0). The standard deviations are indicated. (D) Western blot analysis using anti-EnvZ antibodies. Aliquots (200 μg) of cytoplasmic membrane proteins obtained from wild-type *E*. *coli* K-12 (EnvZ_WT_), EnvZ polyP_P/A(C)_ or *E*. *coli* K-12 Δ*efp* were separated on a SDS polyacrylamide gel. The values for relative band intensities are derived from biological triplicates. The *E*. *coli* K-12 Δ*envZ* strain served as the negative control and was complemented with plasmid-encoded *envZ* (pEnvZ_WT_ [26]) for use as the positive control). (E) Western blot analysis of membrane proteins prepared from wild-type *E*. *coli* K-12 or a Δ*efp* mutant harboring plasmid-encoded *envZ* (pBAD24_EnvZ-FLAG). EnvZ-FLAG was detected with anti-FLAG antibodies. The values for relative band intensities represent biological duplicates.

In the first approach, wild-type *E*. *coli* MG1655, and the isogenic mutants Δ*envZ* and EnvZ_P/A(c)_ (replacement of IPPPL by IAAAL) were grown in media of increasing osmolarity imposed by the addition of NaCl. Outer membrane proteins were isolated and visualized on a Coomassie Blue-stained SDS-urea gel (Fig 3A). In addition, protein band intensities were quantified (Fig 3B). In wild-type cells, OmpC protein levels increase and OmpF levels decrease in response to the increased osmolarity. As expected, strongly reduced and stress-independent OmpC levels were detected in the Δ*envZ* strain. In contrast, the EnvZ_P/A(c)_ variant was associated with high OmpC levels and extremely low OmpF levels under non-stress conditions. In response to osmotic stress only a slight increase in OmpC and decrease in OmpF were observed. This suggests that the EnvZ variant is locked in an ON state [61].

A second approach was used to corroborate this result. This assay is based on a translational *ompC-cfp* fusion in the reporter strain EPB273a [27], and fluorescence was used as the readout to determine the relative level of OmpC (Fig 3C). Cultivation of wild-type cells in the presence of 0.2 M NaCl led to increased OmpC-CFP levels as already described [27]. At higher osmolarities, attained by adding NaCl or sucrose, a further increase in the amount of OmpC-CFP was observed. The isogenic Δ*envZ* mutant produced very low levels of OmpC-CFP protein and responded weakly to osmotic stress. The EnvZ_P/A(c)_ mutant was characterized by significantly higher OmpC-CFP levels in comparison to the wild type under all tested conditions (Fig 3C).

Therefore, we conclude that the IPPPL motif in the EnvZ HAMP domain is important for dimerization of EnvZ and the ability to respond to osmotic stress.

### PolyP_c_ has no effect on the steady-state level or the localization of EnvZ

The translational arrest caused by polyP motifs has the potential to reduce the copy numbers of the corresponding proteins [9]. With only 50 dimers per cell, EnvZ is a low-copy-number receptor [62]. Small changes in molecule number might therefore have significant phenotypic effects. To investigate whether altered EnvZ protein levels, caused by the substitution of alanines for the three prolines in polyP_c_, need to be taken into account in the interpretation of our previous results, we quantitatively analyzed EnvZ by Western blotting. We compared endogenous EnvZ protein levels from *E*. *coli* K-12 wild-type cells to *E*. *coli* K-12 cells producing the EnvZ IAAAL variant but did not find any noteworthy differences (Fig 3D). Moreover, the substituted EnvZ variant was found to be located in the membrane. Due to the replacement of the prolines by alanine, the molecular weight of the protein is expected to decrease slightly (78 Da). However, the corresponding protein band in the Western blot is running about 1,250 Da lower than that of wild-type EnvZ. Presumably this reflects a more compact conformation of EnvZ, which is adopted due to the missing polyP sequence (Fig 2).

EF-P-dependent EnvZ translation only became obvious when *envZ* was expressed from a plasmid under the control of the P_BAD_ promoter (Fig 3E). Under these conditions, wild-type *E*. *coli* produced significantly more EnvZ than the Δ*efp* mutant as revealed by the analysis of the relative band intensities of the Western blot.

These results indicate that the effects seen on dimerization and functionality of EnvZ after replacement of the polyP_c_ motif are not a consequence of an alteration in its copy number or subcellular localization.

### EnvZ in *E*. *coli* harbors a second polyP (VVPPA) motif in the periplasmic domain

**Fig 4:**
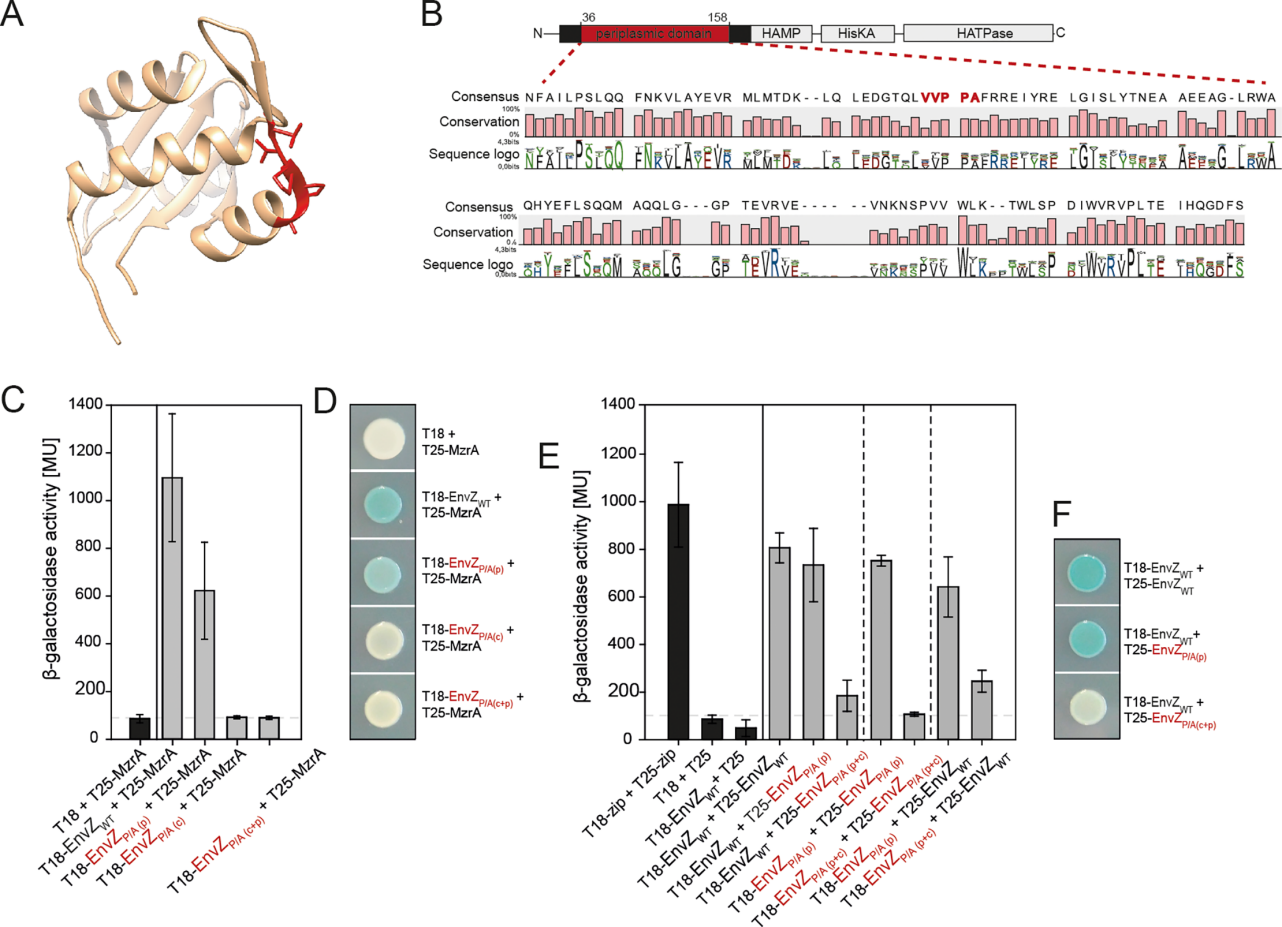
The role of polyP_p_ in the EnvZ-MzrA interaction. (A) The 3D structure of the periplasmic domain of (*E*. *coli* K-12) EnvZ [63]. Proline residues in the PolyP_p_ motif are marked in red. (B) Sequence conservation of the EnvZ periplasmic domain based on multiple sequence alignment of EnvZ homologues (for details see Fig 2). (C) BACTH analysis of the significance of the polyP_p_ and polyP_c_ motifs of EnvZ for its interaction with MzrA, based on the complementation of T25 and T18 adenylate cyclase fragments fused N-terminally to EnvZ variants. β-Galactosidase activities of the reporter strain BTH101 after transformation with plasmids encoding the indicated hybrids and growth in LB-medium. (D) BACTH assay (constructs and cultivation conditions as in (C)) to analyze the effect of polyP_p_ on EnvZ dimerization, quantified by measuring β-galactosidase activities. (E) Determination of β-galactosidase induced blue staining of colonies of BTH101, transformed with the described constructs, and grown on X-Gal/IPTG agar plates for 24 hours. All data are based on biological triplicates. Error bars indicate standard deviations of the mean, and representative plates are shown.

EnvZ contains a second polyP motif (VVPPA) in the periplasmic domain. This motif is one of the medium-to-weak stalling motifs [21]. The periplasmic domain of EnvZ was recently crystallized, and the resolved 3D structure reveals that VVPPA is directed outwards and separates a β-sheet from an α-helix [63] (Fig 4A). Multiple sequence alignment of the 63 species ([52]) that harbor EnvZ reveals that this motif is less conserved than polyP_c_: Val_71_ 30%, Val_72_ 56%, Pro_73_ 59%, Pro_74_ 62%, Ala_75_ 60% (Fig 4B and S1 Fig).

### PolyP_p_ is required for MzrA-EnvZ interaction

Because of its exposed localization within the periplasm, we asked whether the polyP_p_ motif is involved in the interaction of EnvZ with the membrane-integrated MzrA, the modulator protein of EnvZ. Previously, it was shown that two amino acid substitutions in the periplasmic domain of MzrA are sufficient to decrease significantly its capacity to bind to EnvZ [41]. Therefore, we tested for EnvZ-MzrA interaction in vivo by using the BACTH assay.

The interaction between MzrA and EnvZ produced high β-galactosidase activities and blue colonies on indicator plates (Fig 4C and 4D). Substitution of alanines for the two prolines in the polyP_p_ motif led to a decline in the β-galactosidase activities to about 60%, suggesting a decreased affinity of this EnvZ variant for MzrA. In contrast to the polyP_c_ motif, the polyP_p_ motif does not contribute to the dimerization of EnvZ (Fig 4E and 4F). However, replacement of the polyP motif in the HAMP domain abolished the interaction of EnvZ with MzrA completely (β-galactosidase activities of less than 10% of wild type) (Fig 4C). Either the 13 amino acids of MzrA being predicted to be located in the cytoplasm are involved in the interaction with EnvZ or EnvZ dimerization might be a prerequisite for MzrA binding.

### Phylogenetic distribution of the EnvZ polyP_p_ motif and MzrA indicates that they co-evolved

**Fig 5:**
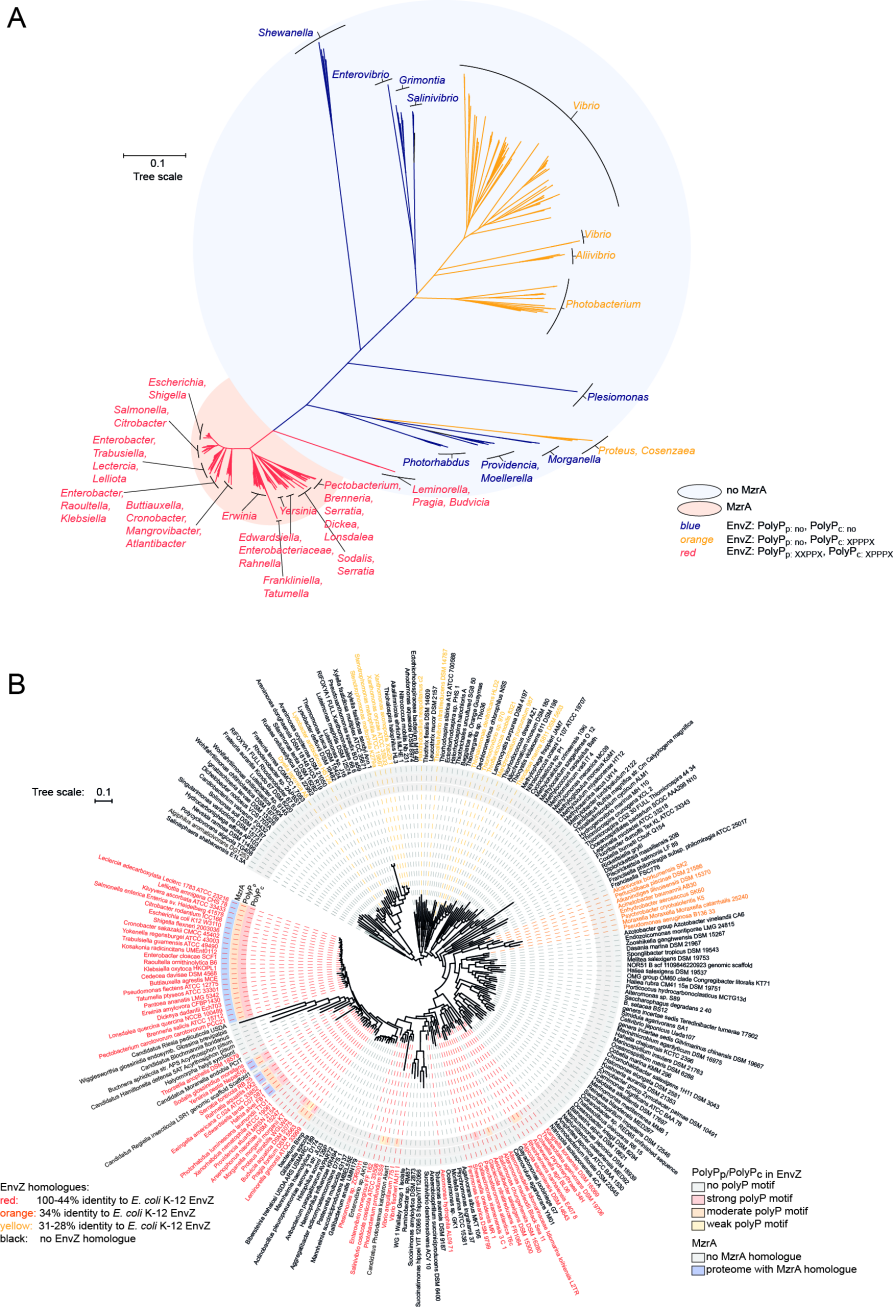
Distribution and evolution of EnvZ and MzrA. (A) Gene tree based on multiple sequence alignment of 793 EnvZ sequences (exhibiting >44% identity to *E*. *coli* K-12 EnvZ) identified by UniProt BLAST search. Strains harboring homologues of *E*. *coli* K-12 MzrA (> 31% sequence identity) appear on a red background or otherwise on a blue background. The presence of polyP_c_ and polyP_p_ is indicated by the branch and letter color. (B) Species subtree of Gammaproteobacteria from a “tree of life” phylogeny [52]. EnvZ homologues of the bacterial species are indicated according to their degree of sequence identity to *E*. *coli* K-12 EnvZ (letter color). The presence of MzrA homologues in some species is marked with a blue background. PolyP motifs are labelled with colored background according to their predicted stalling strength.

Our previous experiments showed that polyP_p_ promotes the interaction of EnvZ with MzrA interaction in *E*. *coli*. Assuming, that MzrA modulates the EnvZ/OmpR system in other microorganisms as well, we examined whether the occurrence of MzrA correlates with the presence of polyP_p_ or at least two consecutive prolines at the corresponding position in EnvZ. In addition, we analyzed the conservation of polyP_c_, which is located within the unstructured connector region of the HAMP domain and is therefore unsuitable for use in common homology models. But taking into account the high level of conservation of the latter motif (Fig 2B) and its effect on the signaling activity of the receptor, we took a more detailed look at its evolution. There are no paralogues of EnvZ within *Escherichia coli*. To visualize the distribution of EnvZ polyP motifs, we collected sequences orthologous to *E*. *coli* K-12 EnvZ from UniProt Microbial Proteins by performing a BLAST search. We identified EnvZ orthologues from 793 organisms with at least 44% sequence identity to the K-12 protein. We constructed a gene tree based on multiple sequence alignment (at least 80% coverage of the shortest protein compared to the longest) (Fig 5A). By NCBI BLAST homology search, we then screened the selected proteomes for orthologues of *E*. *coli* K-12 MzrA (> 31% sequence identity, which was our empirically determined threshold for defining a protein sequence as MzrA). The color code illustrates the presence of polyP_p_ and polyP_c_ in EnvZ as well as the occurrence of a MzrA homologue in the respective organisms (Fig 5A). The polyP_c_ motif (or three consecutive prolines at the equivalent position) is widely distributed. This underlines the importance of the triple proline in the HAMP domain and its higher degree of conservation (Fig 2B). In contrast, polyP_p_ is present only in closely related EnvZ homologues, especially in most representatives of the *Enterobacteriaceae*. Remarkably, MzrA was almost exclusively found in organisms with the EnvZ polyP_p_ motif; the only exceptions were *Ewingella*, *Pragia*, *Leminorella* and *Budvicia*, which exhibit a polyP_p_ motif, but not MzrA. This argues for an evolutionary adaptation of EnvZ to its modulator protein MzrA.

To trace the emergence of the two polyP motifs during evolution, we made use of the above- mentioned phylogenetic species tree of Gammaproteobacteria [52] (Fig 5B). We screened the nodal organisms for orthologues of *E*. *coli* K-12 EnvZ by BLAST homology search and marked them in the tree according to the percentage of sequence identity. In parallel, we screened for the presence of *E*. *coli* K-12 MzrA orthologues (> 31% sequence identity) and depicted the presence of polyP motifs in EnvZ with a color scheme.

Both proline motifs arose late in evolution, and polyP_p_ seems to have evolved together with MzrA. All organisms that encode MzrA also have a polyP_p_ motif in their EnvZ homologues. In addition, we analyzed the development of the putative stalling strength of the proline motifs. Again, we referred to Qi *et al*. for motif classification [21]. There is no change in stalling strength of the medium-to-low periplasmic proline motif, if present, which is compatible with the finding that the selection pressure against polyP motifs increases with the duration of the induced translation arrest. In agreement with this, the expected impact of the cytoplasmic proline motif varies and is only medium or weak in some phylogenetically “older” organisms, like *Budvicia*, *Pragia*, *Salinivibrio*, *Photobacterium*, *Vibrio*, *Alteromonas*, *Algicola* and *Rheinheimera*. In EnvZ both motifs seem to be a consequence of a specialization, in contrast to the expectation that polyP motifs in general developed early during evolution and constantly underlie selection pressure [21, 24].

## Discussion

Here we studied the role of the two polyP motifs in EnvZ of *E*. *coli* K-12. Both motifs are located in exposed structural regions in EnvZ, and we found that they are important for protein-protein interactions. First, we focused on the IPPPL motif, which is thought to induce a strong translation arrest [14]. However, we show here that the strong IPPPL stop motif has almost no effect on the total amount of EnvZ per cell, as the copy number of EnvZ did not change when the prolines were replaced by alanines (Fig 3D). The impact of EF-P only became apparent when the number of *envZ* transcripts was artificially increased by cloning the gene into a plasmid under the control of the P_BAD_ promoter (Fig 3E). Therefore, we conclude that this motif is not primarily required for tight regulation of the EnvZ copy number.

Instead we found that the polyP_c_ motif within the HAMP domain is required for EnvZ dimerization. The HisKA domain of EnvZ is known to be predominantly involved in dimerization, although the whole cytoplasmic homodimerization interface of EnvZ consists of HAMP and HisKA domain [64]. *In vitro* studies revealed, that the HAMP domain is dispensable for dimerization of a truncated HisKA [65]. Its main function is to transduce the extracellular stimulus to the cytoplasmic enzymatic domains of EnvZ [66, 67].

In addition, the enzymatic reaction equilibrium of the EnvZ_P/A(c)_ variant is shifted strongly towards a kinase ON state (Fig 3A–3C). Replacement of the polyP_c_ motif might perturb the homodimeric, four-helical, parallel coiled-coil structure and therefore impairs its conformational flexibility, which locks the receptor in the kinase ON state. Various amino acid substitutions within the HAMP domain of a Tar-EnvZ chimeric receptor lock the enzymatic activity of the protein in the kinase state and cause constitutive expression of OmpC, regardless of the osmotic conditions [68]. This phenotype is also characteristic for the A193V substitution within the EnvZ HAMP domain and is as well observed when the Tar fragment is fused N-terminally directly up- or downstream of the three constitutive prolines in the EnvZ HAMP domain [58, 68]. It has therefore been proposed that these amino acid replacements destabilize the already dynamic HAMP domain and/or interfere with its ability to transmit external stimuli to the HisKA domain [58].

MzrA is known to interact with EnvZ in the periplasm [41]. Unexpectedly, substitution of alanines for the prolines in the polyP_c_ motif completely prevented the EnvZ-MzrA interaction. Williams and Steward suggested nearly 20 years ago that HAMP helix I folds parallel to the membrane surface instead of projecting into the cytoplasm [69], which would allow a physical interaction between MzrA and the polyP_c_ motif. On the other hand, deletion of the first five cytosolic amino acids of MzrA had no effect on the interplay between EnvZ and MzrA [41] and a recent biophysical study showed, that helix I of EnvZ does not interact with a bicelle surface as it is the case for Tar and NarX [70]. Therefore, the decrease in EnvZ-MzrA interaction is more likely to be a consequence of the altered conformation of the EnvZ_P/A(c)_ variant.

Our results demonstrate, that the periplasmic VVPPL motif is involved in the interaction with the membrane-integrated modulator protein MzrA. The importance of the polyP_p_ motif for the interaction with the modulator MzrA is supported by a phylogenetic analysis, which revealed co-evolution between MzrA and polyP_p_ (Fig 5B)

The two polyP motifs in EnvZ were found to be important for protein-protein interactions, and thus join the multitude of proline-containing peptide sequences known to be involved in protein binding. Consecutive prolines are often exposed in protein structures, and their conformational stability allows binding with only small entropic changes. Therefore, proline-rich regions facilitate fast and non-specific binding, in contrast to the slow, but specific binding of globular interaction partners [71]. In accordance with this idea, the binding affinity of numerous proteins, such as human salivary proteins [72] or the actin-binding protein from *Dictyostelium discoideum* [73], has previously been linked to their proline-rich sequence motifs.

Given the broad range of protein families that exhibit polyP stalling motifs, their role and function are presumably diverse. While polyP motifs are important for the regulation of the copy number of the pH sensor CadC [9], they promote protein-protein interactions in the osmolarity and pH sensor EnvZ. PolyP motifs occur in various functional domains in other HKs and their individual roles remain to be experimentally clarified.

## Supporting information

S1 Fig Sequence alignment, based on 63 EnvZ homologues (exhibiting >44% sequence identity to *E*. *coli* K-12 EnvZ) from a phylogenetic tree of representative Gammaproteobacteria [52]. (PDF)
