# Supplementary material for: The role of polyproline motifs in the histidine kinase EnvZ
Source: PLoS One. 2018 Jun 28;13(6):e0199782. doi: 10.1371/journal.pone.0199782 (PMC6023141; doi:10.1371/journal.pone.0199782)

Supporting Information

The role of polyproline motifs in the histidine kinase EnvZ

Magdalena Motz[1] and Kirsten Jung[1*]

[1] Center for Integrated Protein Science Munich at the Department of Biology I, Microbiology, Ludwig-Maximilians-Universität München, D-82152 Martinsried, Germany

* Corresponding author

E-mail: jung@lmu.de

Fig S1: Sequence alignment, based on 63 EnvZ homologues (exhibiting >44% sequence identity to *E. coli* K-12 EnvZ) from a phylogenetic tree of representative Gammaproteobacteria (Hug LA, Baker BJ, Anantharaman K, Brown CT, Probst AJ, Castelle CJ, *et al*. A new view of the tree of life. Nature microbiology. 2016;1:16048.).

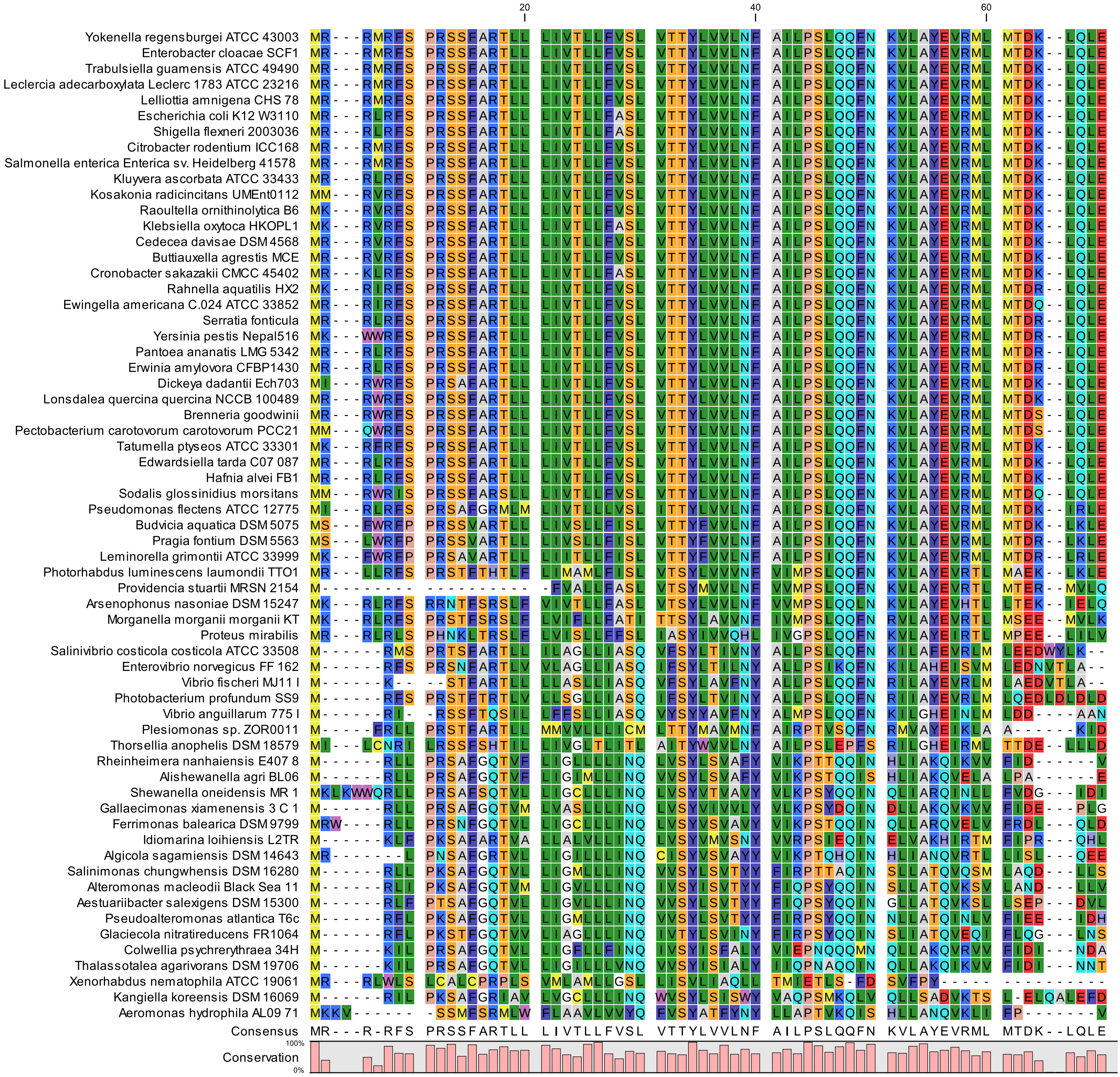
20
40
60
Yokenella regensburgei ATCC 43003
Enterobacter cloacae SCF1
Trabulsiella guamensis ATCC 49490
Leclercia adecarboxylata Leclerc 1783 ATCC 23216
Lelliottia amnigena CHS 78
Escherichia coli K12 W3110
Shigella flexneri 2003036
Citrobacter rodentium ICC168
Salmonella enterica Enterica sv. Heidelberg 41578
Kluyvera ascorbata ATCC 33433
Kosakonia radicincitans UMEnt0112
Raoultella ornithinolytica B6
Klebsiella oxytoca HKOPL1
Cedecea davisae DSM 4568
Buttiauxella agrestis MCE
Cronobacter sakazakii CMCC 45402
Rahnella aquatilis HX2
Ewingella americana C.024 ATCC 33852
Serratia fonticula
Yersinia pestis Nepal516
Pantoea ananatis LMG 5342
Erwinia amylovora CFBP1430
Dickeya dadantii Ech703
Lonsdalea quercina quercina NCCB 100489
Brenneria goodwinii
Pectobacterium carotovorum carotovorum PCC21
Tatumella ptyseos ATCC 33301
Edwardsiella tarda C07 087
Hafnia alvei FB1
Sodalis glossinidius morsitans
Pseudomonas flectens ATCC 12775
Budvicia aquatica DSM 5075
Pragia fontium DSM 5563
Leminorella grimontii ATCC 33999
Photorhabdus luminescens laumondii TTO1
Providencia stuartii MRSN 2154
Arsenophonus nasoniae DSM 15247
Morganella morganii morganii KT
Proteus mirabilis
Salinivibrio costicola costicola ATCC 33508
Enterovibrio norvegicus FF 162
Vibrio fischeri MJ11 I
Photobacterium profundum SS9
Vibrio anguillarum 775 I
Plesiomonas sp. ZOR0011
Thorsellia anophelis DSM 18579
Rheinheimera nanhaiensis E407 8
Alishewanella agri BL06
Shewanella oneidensis MR 1
Gallaecimonas xiamenensis 3 C 1
Ferrimonas balearica DSM 9799
Idiomarina loihiensis L2TR
Algicola sagamiensis DSM 14643
Salinimonas chungwhensis DSM 16280
Alteromonas macleodii Black Sea 11
Aestuariibacter salexigens DSM 15300
Pseudoalteromonas atlantica T6c
Glaciecola nitratireducens FR1064
Colwellia psychrerythraea 34H
Thalassotalea agarivorans DSM 19706
Xenorhabdus nematophila ATCC 19061
Kangiella koreensis DSM 16069
Aeromonas hydrophila AL09 71
Consensus
MR - - - R - RFS PRSSFARTLL LIVTLLFVSL VTTYLVVLNF AILPSLQQFN KVLAYEVRML MTDK - - LQLE
Conservation
100%
0%

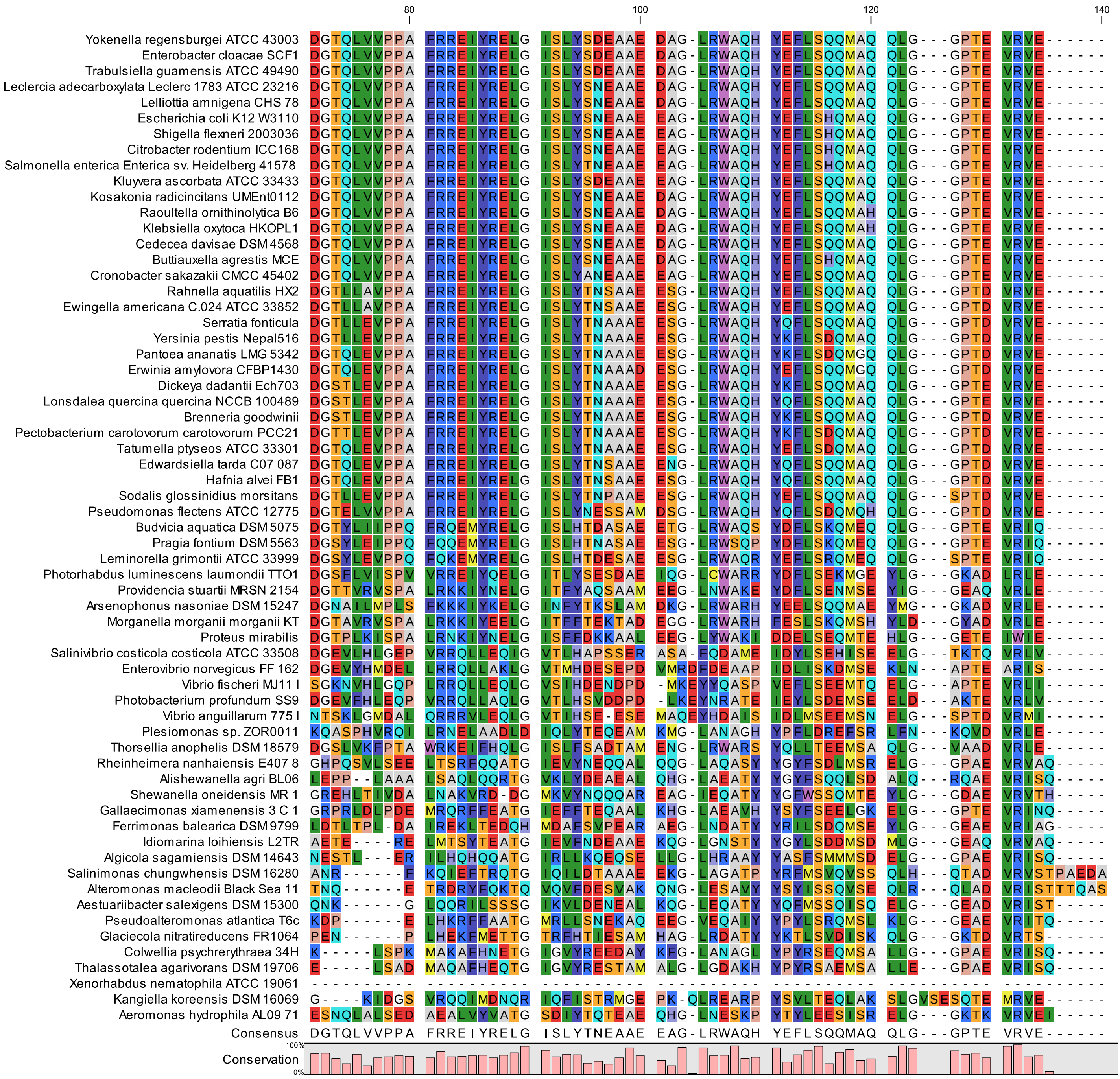
80
100
120
140
Yokenella regensburgei ATCC 43003
Enterobacter cloacae SCF1
Trabulsiella guamensis ATCC 49490
Leclercia adecarboxylata Leclerc 1783 ATCC 23216
Lelliottia amnigena CHS 78
Escherichia coli K12 W3110
Shigella flexneri 2003036
Citrobacter rodentium ICC168
Salmonella enterica Enterica sv. Heidelberg 41578
Kluyvera ascorbata ATCC 33433
Kosakonia radicincitans UMEnt0112
Raoultella ornithinolytica B6
Klebsiella oxytoca HKOPL1
Cedecea davisae DSM 4568
Buttiauxella agrestis MCE
Cronobacter sakazakii CMCC 45402
Rahnella aquatilis HX2
Ewingella americana C.024 ATCC 33852
Serratia fonticula
Yersinia pestis Nepal516
Pantoea ananatis LMG 5342
Erwinia amylovora CFBP1430
Dickeya dadantii Ech703
Lonsdalea quercina quercina NCCB 100489
Brenneria goodwinii
Pectobacterium carotovorum carotovorum PCC21
Tatumella ptyseos ATCC 33301
Edwardsiella tarda C07 087
Hafnia alvei FB1
Sodalis glossinidius morsitans
Pseudomonas flectens ATCC 12775
Budvicia aquatica DSM 5075
Pragia fontium DSM 5563
Leminorella grimontii ATCC 33999
Photorhabdus luminescens laumondii TTO1
Providencia stuartii MRSN 2154
Arsenophonus nasoniae DSM 15247
Morganella morganii morganii KT
Proteus mirabilis
Salinivibrio costicola costicola ATCC 33508
Enterovibrio norvegicus FF 162
Vibrio fischeri MJ11 I
Photobacterium profundum SS9
Vibrio anguillarum 775 I
Plesiomonas sp. ZOR0011
Thorsellia anophelis DSM 18579
Rheinheimera nanhaiensis E407 8
Alishewanella agri BL06
Shewanella oneidensis MR 1
Gallaecimonas xiamenensis 3 C 1
Ferrimonas balearica DSM 9799
Idiomarina loihiensis L2TR
Algicola sagamiensis DSM 14643
Salinimonas chungwhensis DSM 16280
Alteromonas macleodii Black Sea 11
Aestuariibacter salexigens DSM 15300
Pseudoalteromonas atlantica T6c
Glaciecola nitratireducens FR1064
Colwellia psychrerythraea 34H
Thalassotalea agarivorans DSM 19706
Xenorhabdus nematophila ATCC 19061
Kangiella koreensis DSM 16069
Aeromonas hydrophila AL09 71
Consensus
DGTQLVVPPA FRREIYRELG ISLYTNEAAE EAG-LRWAQH YEFLSQQMAQ QLG---GPTE VRVE------
100%
Conservation
0%

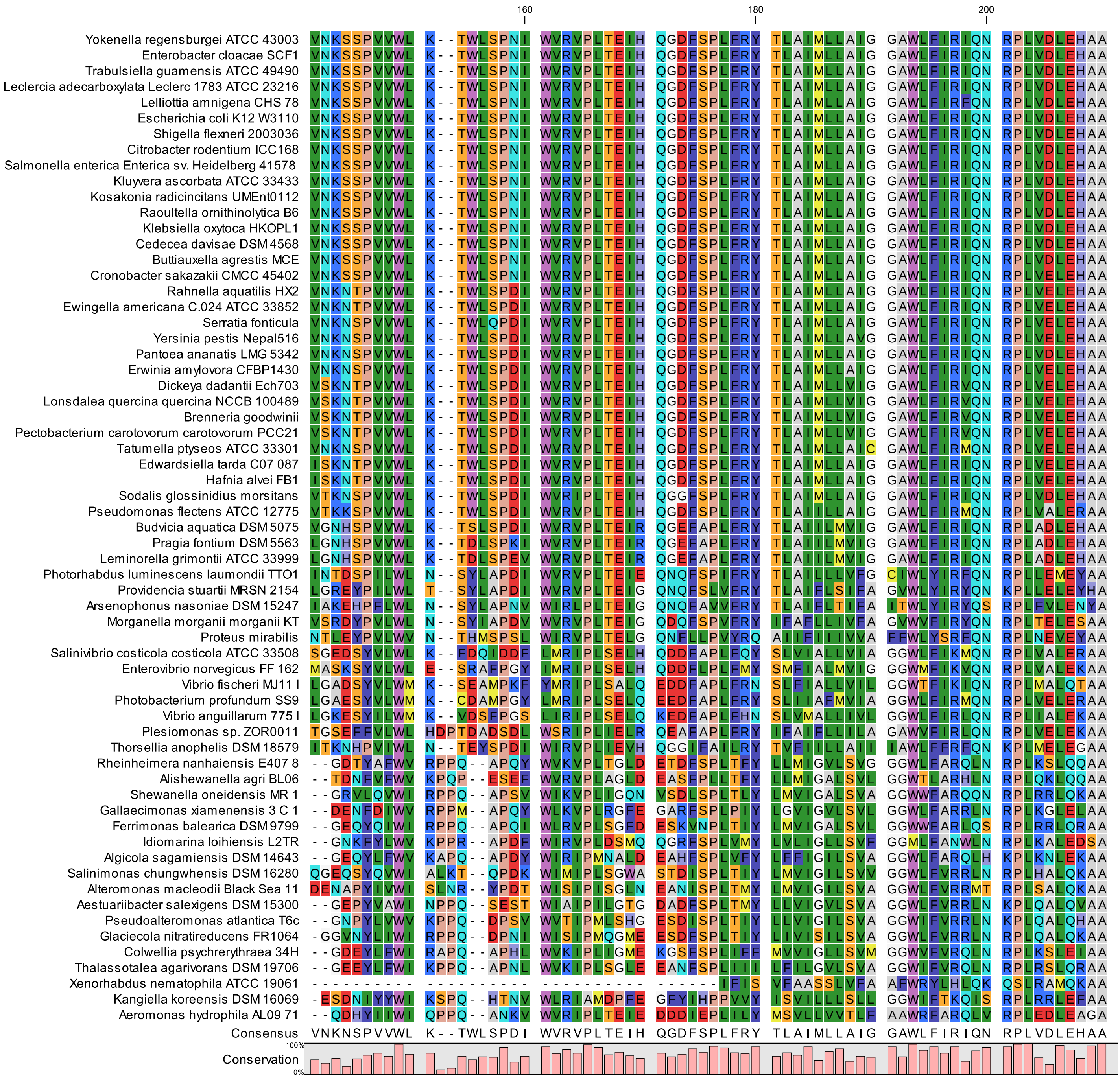
160
180
200
Yokenella regensburgei ATCC 43003
Enterobacter cloacae SCF1
Trabulsiella guamensis ATCC 49490
Leclercia adecarboxylata Leclerc 1783 ATCC 23216
Lelliottia amnigena CHS 78
Escherichia coli K12 W3110
Shigella flexneri 2003036
Citrobacter rodentium ICC168
Salmonella enterica Enterica sv. Heidelberg 41578
Kluyvera ascorbata ATCC 33433
Kosakonia radicincitans UMEnt0112
Raoultella ornithinolytica B6
Klebsiella oxytoca HKOPL1
Cedecea davisae DSM 4568
Buttiauxella agrestis MCE
Cronobacter sakazakii CMCC 45402
Rahnella aquatilis HX2
Ewingella americana C.024 ATCC 33852
Serratia fonticula
Yersinia pestis Nepal516
Pantoea ananatis LMG 5342
Erwinia amylovora CFBP1430
Dickeya dadantii Ech703
Lonsdalea quercina quercina NCCB 100489
Brenneria goodwinii
Pectobacterium carotovorum carotovorum PCC21
Tatumella ptyseos ATCC 33301
Edwardsiella tarda C07 087
Hafnia alvei FB1
Sodalis glossinidius morsitans
Pseudomonas flectens ATCC 12775
Budvicia aquatica DSM 5075
Pragia fontium DSM 5563
Leminorella grimontii ATCC 33999
Photorhabdus luminescens laumondii TTO1
Providencia stuartii MRSN 2154
Arsenophonus nasoniae DSM 15247
Morganella morganii morganii KT
Proteus mirabilis
Salinivibrio costicola costicola ATCC 33508
Enterovibrio norvegicus FF 162
Vibrio fischeri MJ11 I
Photobacterium profundum SS9
Vibrio anguillarum 775 I
Plesiomonas sp. ZOR0011
Thorsellia anophelis DSM 18579
Rheinheimera nanhaiensis E407 8
Alishewanella agri BL06
Shewanella oneidensis MR 1
Gallaecimonas xiamenensis 3 C 1
Ferrimonas balearica DSM 9799
Idiomarina loihiensis L2TR
Algicola sagamiensis DSM 14643
Salinimonas chungwhensis DSM 16280
Alteromonas macleodii Black Sea 11
Aestuariibacter salexigens DSM 15300
Pseudoalteromonas atlantica T6c
Glaciecola nitratireducens FR1064
Colwellia psychrerythraea 34H
Thalassotalea agarivorans DSM 19706
Xenorhabdus nematophila ATCC 19061
Kangiella koreensis DSM 16069
Aeromonas hydrophila AL09 71
Consensus VNKNSPVVWL K--TWLSPDI WVRVPLTEIH QGDFSPLFRY TLAIMLLAIG GAWLFIRIQN RPLVDLEHAA
Conservation
100%
0%

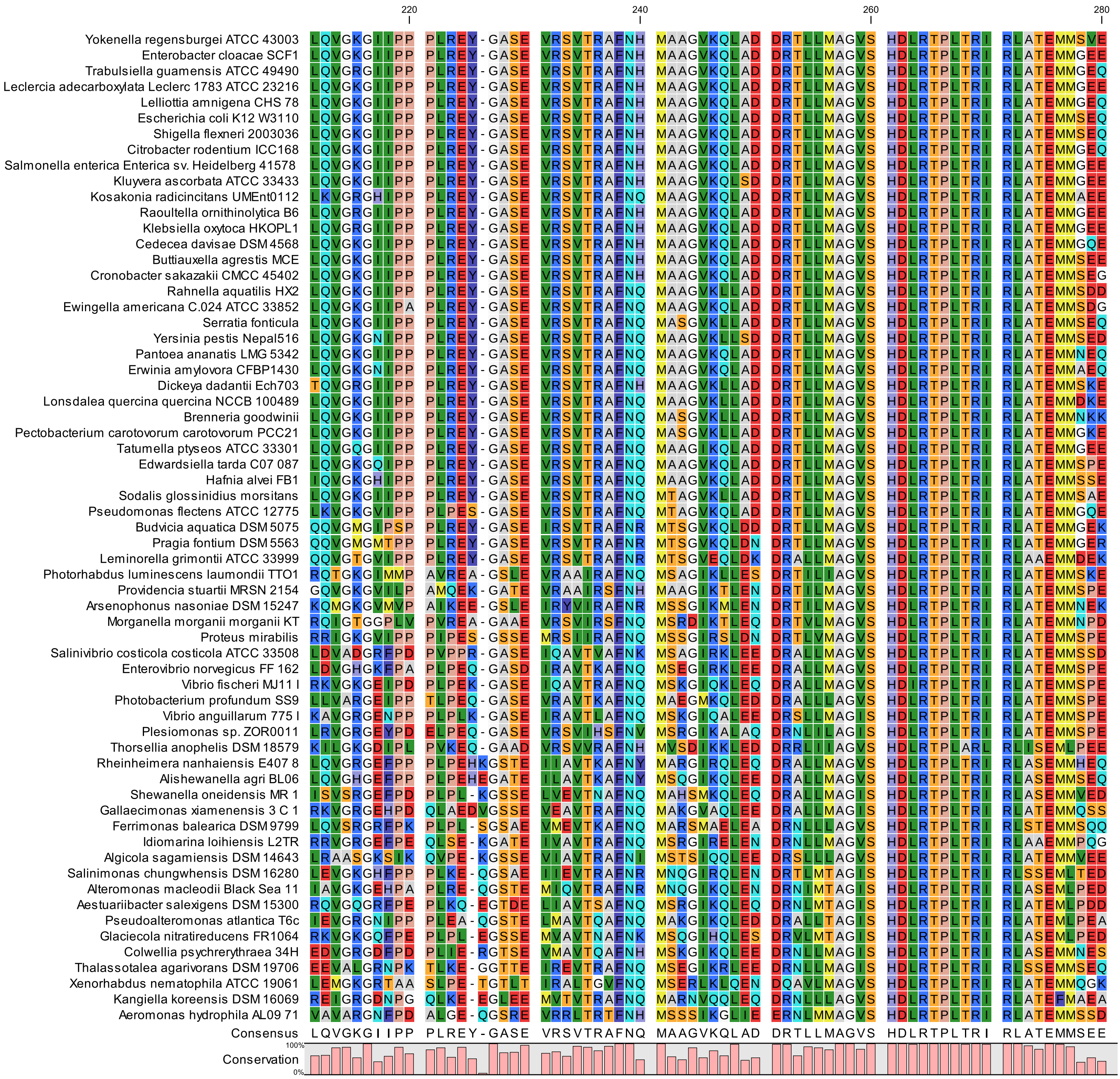

220
240
260
280
Yokenella regensburgei ATCC 43003
Enterobacter cloacae SCF1
Trabulsiella guamensis ATCC 49490
Leclercia adecarboxylata Leclerc 1783 ATCC 23216
Lelliottia amnigena CHS 78
Escherichia coli K12 W3110
Shigella flexneri 2003036
Citrobacter rodentium ICC168
Salmonella enterica Enterica sv. Heidelberg 41578
Kluyvera ascorbata ATCC 33433
Kosakonia radicincitans UMEnt0112
Raoultella ornithinolytica B6
Klebsiella oxytoca HKOPL1
Cedecea davisae DSM 4568
Buttiauxella agrestis MCE
Cronobacter sakazakii CMCC 45402
Rahnella aquatilis HX2
Ewingella americana C.024 ATCC 33852
Serratia fonticula
Yersinia pestis Nepal516
Pantoea ananatis LMG 5342
Erwinia amylovora CFBP1430
Dickeya dadantii Ech703
Lonsdalea quercina quercina NCCB 100489
Brenneria goodwinii
Pectobacterium carotovorum carotovorum PCC21
Tatumella ptyseos ATCC 33301
Edwardsiella tarda C07 087
Hafnia alvei FB1
Sodalis glossinidius morsitans
Pseudomonas flectens ATCC 12775
Budvicia aquatica DSM 5075
Pragia fontium DSM 5563
Leminorella grimontii ATCC 33999
Photorhabdus luminescens laumondii TTO1
Providencia stuartii MRSN 2154
Arsenophonus nasoniae DSM 15247
Morganella morganii morganii KT
Proteus mirabilis
Salinivibrio costicola costicola ATCC 33508
Enterovibrio norvegicus FF 162
Vibrio fischeri MJ11 I
Photobacterium profundum SS9
Vibrio anguillarum 775 I
Plesiomonas sp. ZOR0011
Thorsellia anophelis DSM 18579
Rheinheimera nanhaiensis E407 8
Alishewanella agri BL06
Shewanella oneidensis MR 1
Gallaecimonas xiamenensis 3 C 1
Ferrimonas balearica DSM 9799
Idiomarina loihiensis L2TR
Algicola sagamiensis DSM 14643
Salinimonas chungwhensis DSM 16280
Alteromonas macleodii Black Sea 11
Aestuariibacter salexigens DSM 15300
Pseudoalteromonas atlantica T6c
Glaciecola nitratireducens FR1064
Colwellia psychrerythraea 34H
Thalassotalea agarivorans DSM 19706
Xenorhabdus nematophila ATCC 19061
Kangiella koreensis DSM 16069
Aeromonas hydrophila AL09 71
Consensus
LQVGKGIIPP PLREY-GASE VRSVTRAFNQ MAAGVKQLAD DRTLLMAGVS HDLRTPLTRI RLATEMMSEE
Conservation
100%
0%

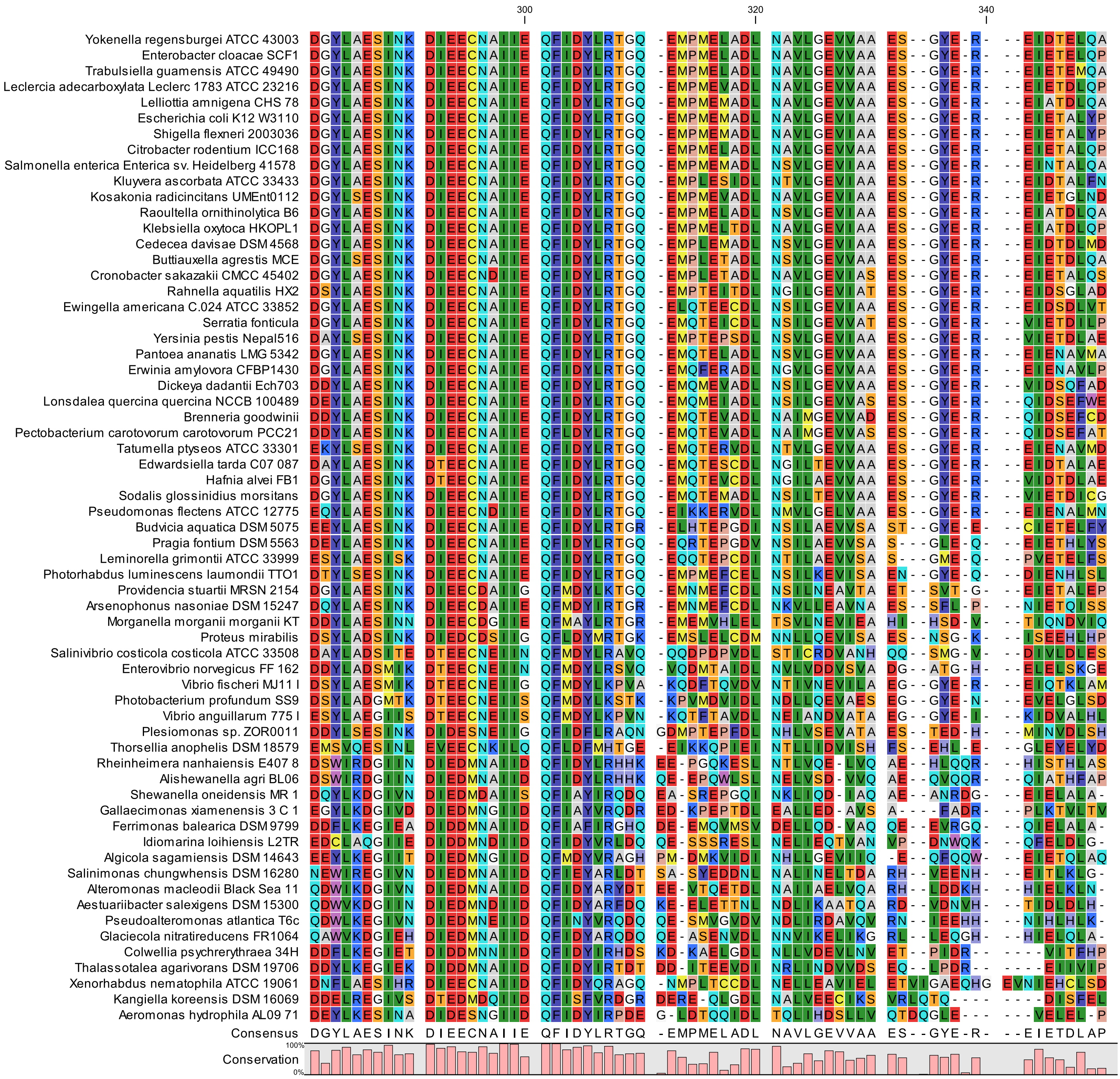
300
320
340
Yokenella regensburgei ATCC 43003
Enterobacter cloacae SCF1
Trabulsiella guamensis ATCC 49490
Leclercia adecarboxylata Leclerc 1783 ATCC 23216
Lelliottia amnigena CHS 78
Escherichia coli K12 W3110
Shigella flexneri 2003036
Citrobacter rodentium ICC168
Salmonella enterica Enterica sv. Heidelberg 41578
Kluyvera ascorbata ATCC 33433
Kosakonia radicincitans UMEnt0112
Raoultella ornithinolytica B6
Klebsiella oxytoca HKOPL1
Cedecea davisae DSM 4568
Buttiauxella agrestis MCE
Cronobacter sakazakii CMCC 45402
Rahnella aquatilis HX2
Ewingella americana C.024 ATCC 33852
Serratia fonticula
Yersinia pestis Nepal516
Pantoea ananatis LMG 5342
Erwinia amylovora CFBP1430
Dickeya dadantii Ech703
Lonsdalea quercina quercina NCCB 100489
Brenneria goodwinii
Pectobacterium carotovorum carotovorum PCC21
Tatumella ptyseos ATCC 33301
Edwardsiella tarda C07 087
Hafnia alvei FB1
Sodalis glossinidius morsitans
Pseudomonas flectens ATCC 12775
Budvicia aquatica DSM 5075
Pragia fontium DSM 5563
Leminorella grimontii ATCC 33999
Photorhabdus luminescens laumondii TTO1
Providencia stuartii MRSN 2154
Arsenophonus nasoniae DSM 15247
Morganella morganii morganii KT
Proteus mirabilis
Salinivibrio costicola costicola ATCC 33508
Enterovibrio norvegicus FF 162
Vibrio fischeri MJ11 I
Photobacterium profundum SS9
Vibrio anguillarum 775 I
Plesiomonas sp. ZOR0011
Thorsellia anophelis DSM 18579
Rheinheimera nanhaiensis E407 8
Alishewanella agri BL06
Shewanella oneidensis MR 1
Gallaecimonas xiamenensis 3 C 1
Ferrimonas balearica DSM 9799
Idiomarina loihiensis L2TR
Algicola sagamiensis DSM 14643
Salinimonas chungwhensis DSM 16280
Alteromonas macleodii Black Sea 11
Aestuariibacter salexigens DSM 15300
Pseudoalteromonas atlantica T6c
Glaciecola nitratireducens FR1064
Colwellia psychrerythraea 34H
Thalassotalea agarivorans DSM 19706
Xenorhabdus nematophila ATCC 19061
Kangiella koreensis DSM 16069
Aeromonas hydrophila AL09 71
Consensus DGYLAESINK DIEECNAIIE QFIDYLRTGQ -EMPMELADL NAVLGEVVAA ES--GYE-R- --EIETDLAP
Conservation
100%
0%

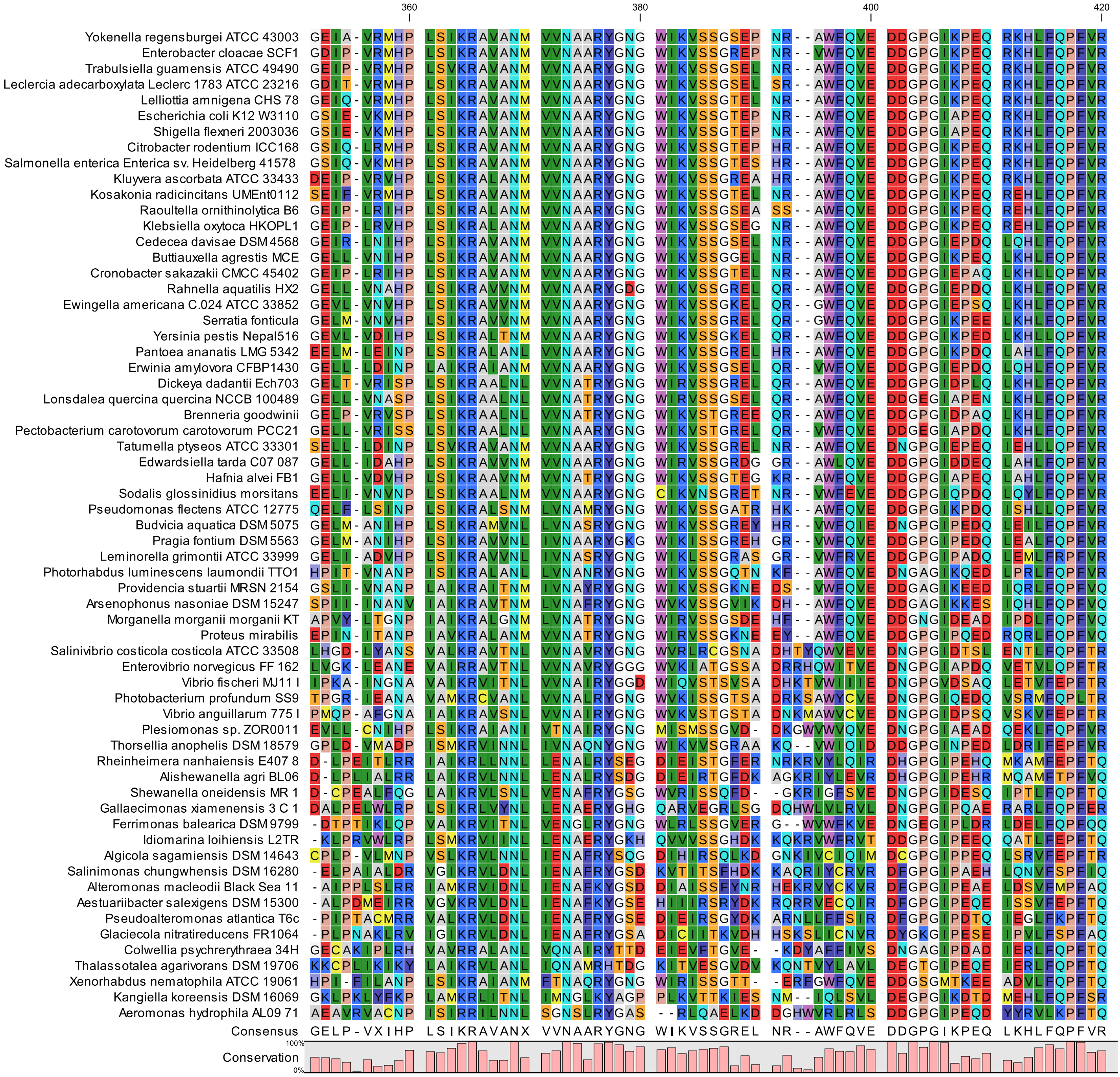
360
380
400
420
Yokenella regensburgei ATCC 43003
Enterobacter cloacae SCF1
Trabulsiella guamensis ATCC 49490
Leclercia adecarboxylata Leclerc 1783 ATCC 23216
Lelliottia amnigena CHS 78
Escherichia coli K12 W3110
Shigella flexneri 2003036
Citrobacter rodentium ICC168
Salmonella enterica Enterica sv. Heidelberg 41578
Kluyvera ascorbata ATCC 33433
Kosakonia radicincitans UMEnt0112
Raoultella ornithinolytica B6
Klebsiella oxytoca HKOPL1
Cedecea davisae DSM 4568
Buttiauxella agrestis MCE
Cronobacter sakazakii CMCC 45402
Rahnella aquatilis HX2
Ewingella americana C.024 ATCC 33852
Serratia fonticula
Yersinia pestis Nepal516
Pantoea ananatis LMG 5342
Erwinia amylovora CFBP1430
Dickeya dadantii Ech703
Lonsdalea quercina quercina NCCB 100489
Brenneria goodwinii
Pectobacterium carotovorum carotovorum PCC21
Tatumella ptyseos ATCC 33301
Edwardsiella tarda C07 087
Hafnia alvei FB1
Sodalis glossinidius morsitans
Pseudomonas flectens ATCC 12775
Budvicia aquatica DSM 5075
Pragia fontium DSM 5563
Leminorella grimontii ATCC 33999
Photorhabdus luminescens laumondii TTO1
Providencia stuartii MRSN 2154
Arsenophonus nasoniae DSM 15247
Morganella morganii morganii KT
Proteus mirabilis
Salinivibrio costicola costicola ATCC 33508
Enterovibrio norvegicus FF 162
Vibrio fischeri MJ11 I
Photobacterium profundum SS9
Vibrio anguillarum 775 I
Plesiomonas sp. ZOR0011
Thorsellia anophelis DSM 18579
Rheinheimera nanhaiensis E407 8
Alishewanella agri BL06
Shewanella oneidensis MR 1
Gallaecimonas xiamenensis 3 C 1
Ferrimonas balearica DSM 9799
Idiomarina loihiensis L2TR
Algicola sagamiensis DSM 14643
Salinimonas chungwhensis DSM 16280
Alteromonas macleodii Black Sea 11
Aestuariibacter salexigens DSM 15300
Pseudoalteromonas atlantica T6c
Glaciecola nitratireducens FR1064
Colwellia psychrerythraea 34H
Thalassotalea agarivorans DSM 19706
Xenorhabdus nematophila ATCC 19061
Kangiella koreensis DSM 16069
Aeromonas hydrophila AL09 71
Consensus GELP-VXIHP LSIKRAVANX VVNAARYGNG WIKVSSGREL NR--AWFQVE DDGPGIKPEQ LKHLFQPFVR
Conservation
100%
0%

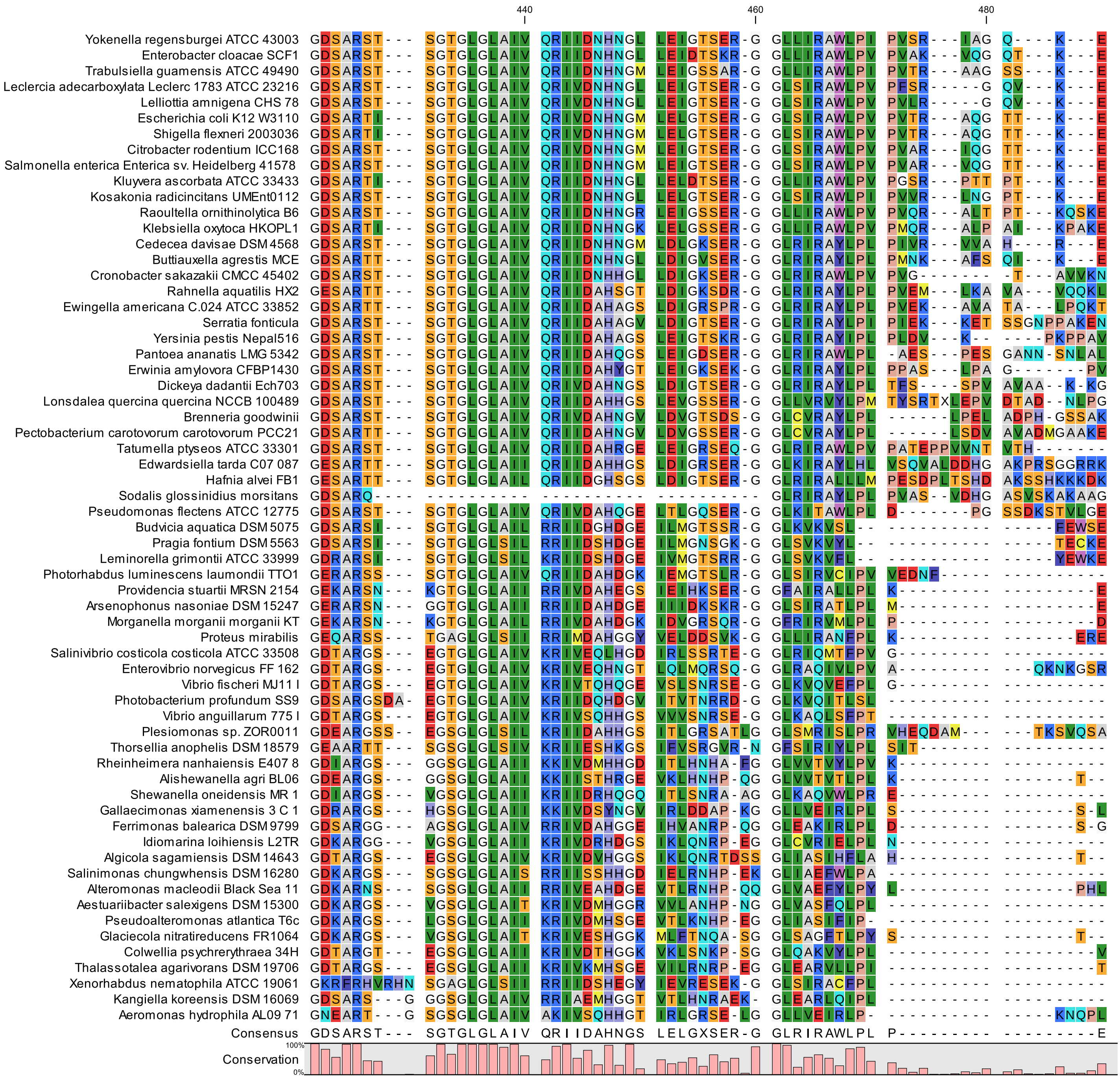
440
460
480
Yokenella regensburgei ATCC 43003
Enterobacter cloacae SCF1
Trabulsiella guamensis ATCC 49490
Leclercia adecarboxylata Leclerc 1783 ATCC 23216
Lelliottia amnigena CHS 78
Escherichia coli K12 W3110
Shigella flexneri 2003036
Citrobacter rodentium ICC168
Salmonella enterica Enterica sv. Heidelberg 41578
Kluyvera ascorbata ATCC 33433
Kosakonia radicincitans UMEnt0112
Raoultella ornithinolytica B6
Klebsiella oxytoca HKOPL1
Cedecea davisae DSM 4568
Buttiauxella agrestis MCE
Cronobacter sakazakii CMCC 45402
Rahnella aquatilis HX2
Ewingella americana C.024 ATCC 33852
Serratia fonticula
Yersinia pestis Nepal516
Pantoea ananatis LMG 5342
Erwinia amylovora CFBP1430
Dickeya dadantii Ech703
Lonsdalea quercina quercina NCCB 100489
Brenneria goodwinii
Pectobacterium carotovorum carotovorum PCC21
Tatumella ptyseos ATCC 33301
Edwardsiella tarda C07 087
Hafnia alvei FB1
Sodalis glossinidius morsitans
Pseudomonas flectens ATCC 12775
Budvicia aquatica DSM 5075
Pragia fontium DSM 5563
Leminorella grimontii ATCC 33999
Photorhabdus luminescens laumondii TTO1
Providencia stuartii MRSN 2154
Arsenophonus nasoniae DSM 15247
Morganella morganii morganii KT
Proteus mirabilis
Salinivibrio costicola costicola ATCC 33508
Enterovibrio norvegicus FF 162
Vibrio fischeri MJ11 I
Photobacterium profundum SS9
Vibrio anguillarum 775 I
Plesiomonas sp. ZOR0011
Thorsellia anophelis DSM 18579
Rheinheimera nanhaiensis E407 8
Alishewanella agri BL06
Shewanella oneidensis MR 1
Gallaecimonas xiamenensis 3 C 1
Ferrimonas balearica DSM 9799
Idiomarina loihiensis L2TR
Algicola sagamiensis DSM 14643
Salinimonas chungwhensis DSM 16280
Alteromonas macleodii Black Sea 11
Aestuariibacter salexigens DSM 15300
Pseudoalteromonas atlantica T6c
Glaciecola nitratireducens FR1064
Colwellia psychrerythraea 34H
Thalassotalea agarivorans DSM 19706
Xenorhabdus nematophila ATCC 19061
Kangiella koreensis DSM 16069
Aeromonas hydrophila AL09 71
Consensus GDSARST--- SGTGLGLAIV QRIIDAHNGS LELGXSER-G GLRIRAWLPL P--------- ---------E
Conservation
100%
0%

Yokenella regensburgei ATCC 43003	- - - S	449
Enterobacter cloacae SCF1	- - - S	450
Trabulsiella guamensis ATCC 49490	- - - S	450
Leclercia adecarboxylata Leclerc 1783 ATCC 23216	- - - S	448
Lelliottia amnigena CHS 78	- - - S	448
Escherichia coli K12 W3110	- - - G	450
Shigella flexneri 2003036	- - - G	450
Citrobacter rodentium ICC168	- - - A	450
Salmonella enterica Enterica sv. Heidelberg 41578	- - - A	450
Kluyvera ascorbata ATCC 33433	- - - S	450
Kosakonia radicincitans UMEnt0112	- - - A	450
Raoultella ornithinolytica B6	- - - S	453
Klebsiella oxytoca HKOPL1	- - - I	453
Cedecea davisae DSM 4568	- - - S	449
Buttiauxella agrestis MCE	- - - S	450
Cronobacter sakazakii CMCC 45402	- - - S	448
Rahnella aquatilis HX2	- - - V	453
Ewingella americana C.024 ATCC 33852	- - - V	453
Serratia fonticula	- - - A	456
Yersinia pestis Nepal516	- - - A	450
Pantoea ananatis LMG 5342	- - - S	454
Erwinia amylovora CFBP1430	- - - A	449
Dickeya dadantii Ech703	- - - G	452
Lonsdalea quercina quercina NCCB 100489	- - - A	457
Brenneria goodwinii	- - - A	452
Pectobacterium carotovorum carotovorum PCC21	- - - S	453
Tatumella ptyseos ATCC 33301	- - - -	451
Edwardsiella tarda C07 087	E - H S	461
Hafnia alvei FB1	E S H H	462
Sodalis glossinidius morsitans	K S A D	430
Pseudomonas flectens ATCC 12775	N V R E	455
Budvicia aquatica DSM 5075	N N - -	443
Pragia fontium DSM 5563	N D L K	444
Leminorella grimontii ATCC 33999	K E - E	443
Photorhabdus luminescens laumondii TTO1	- - - -	443
Providencia stuartii MRSN 2154	- K E E	426
Arsenophonus nasoniae DSM 15247	- K - -	441
Morganella morganii morganii KT	D R D D	444
Proteus mirabilis	D E D D	446
Salinivibrio costicola costicola ATCC 33508	- - - K	439
Enterovibrio norvegicus FF 162	K E M R	450
Vibrio fischeri MJ11 I	- - - -	434
Photobacterium profundum SS9	- - V K	443
Vibrio anguillarum 775 I	- - - K	434
Plesiomonas sp. ZOR0011	K K T S	456
Thorsellia anophelis DSM 18579	- S S K	444
Rheinheimera nanhaiensis E407 8	- - - -	434
Alishewanella agri BL06	- - E L	435
Shewanella oneidensis MR 1	- - - -	438
Gallaecimonas xiamenensis 3 C 1	H P V S	442
Ferrimonas balearica DSM 9799	E - - -	437
Idiomarina loihiensis L2TR	Q Q R Q	436
Algicola sagamiensis DSM 14643	- - - -	434
Salinimonas chungwhensis DSM 16280	- - - -	436
Alteromonas macleodii Black Sea 11	H Q K D	444
Aestuariibacter salexigens DSM 15300	- N Q P	432
Pseudoalteromonas atlantica T6c	H T C P	432
Glaciecola nitratireducens FR1064	- - - -	431
Colwellia psychrerythraea 34H	F K - -	430
Thalassotalea agarivorans DSM 19706	R E R S	434
Xenorhabdus nematophila ATCC 19061	- N T K	342
Kangiella koreensis DSM 16069	- - - H	434
Aeromonas hydrophila AL09 71	E W I D	436
Consensus	- - - S	

Conservation
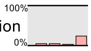

Supplement: S1 Fig — (PDF) [file pone.0199782.s001.pdf]
